# Canonical and Non-Canonical Regulators of Lipid Metabolism in the Liver and the “Liver–Other Organs” Axes

**DOI:** 10.3390/ijms27198614

**Published:** 2026-09-26

**Authors:** Stanislav Kotlyarov, Anna Kotlyarova, Roberto Catanzaro, Francesco Marotta

**Affiliations:** 1 Department of Nursing, Ryazan State Medical University, 390026 Ryazan, Russia; 2 Department of Pharmaceutical Technology and Pharmacy Practice Administration, Ryazan State Medical University, 390026 Ryazan, Russia; kaa.rz@yandex.ru; 3 Department of Clinical and Experimental Medicine, University of Catania, 95123 Catania, Italy; rcatanza@unict.it; 4 Regenera R&D International for Aging Intervention, 20154 Milano, Italy

**Keywords:** metabolic dysfunction-associated steatotic liver disease, hepatic lipid metabolism, fibroblast growth factors, adiponectin, leptin, apolipoprotein C-III, serotonin, dopamine, insulin resistance, cardiovascular–kidney–metabolic syndrome

## Abstract

Metabolic dysfunction-associated steatotic liver disease (MASLD) is the most common chronic liver disease and is closely associated with obesity, insulin resistance, and the recently conceptualized cardiovascular–kidney–metabolic syndrome. For decades, the pathogenesis of steatosis has been viewed primarily through the lens of insulin and the canonical transcriptional programs it regulates—sterol regulatory element-binding protein 1c, carbohydrate response element-binding protein, peroxisome proliferator-activated receptors, and AMP-activated protein kinase. At the same time, recent data point to the existence of a complex network of non-canonical regulators that, independently of insulin, significantly modulate hepatic lipid metabolism. This review systematizes information on the fibroblast growth factors FGF21 and FGF19, adipokines (adiponectin, leptin), apolipoprotein C-III, as well as the monoamine neurotransmitters serotonin and dopamine. These molecules act within the interorgan axes “liver–adipose tissue,” “gut–liver,” “brain–liver,” and “liver–spleen,” coordinating lipolysis, gluconeogenesis, very-low-density lipoprotein secretion, inflammation, and fibrogenesis. Disruption of the dynamic balance between canonical and non-canonical regulators is central to the progression from simple steatosis to steatohepatitis and fibrosis. The clinical translation of this concept has already begun: the thyroid hormone receptor β agonist resmetirom and the glucagon-like peptide-1 (GLP-1) receptor agonist semaglutide have received regulatory approval for specific categories of patients with noncirrhotic metabolic dysfunction-associated steatohepatitis and fibrosis, while FGF21 and FGF19 analogs, the dual glucose-dependent insulinotropic polypeptide (GIP)/GLP-1 agonist tirzepatide, and agents targeting apolipoprotein C-III are at various stages of clinical development or are being used for related metabolic indications. Expanding the insulin-centric paradigm to include non-canonical axes opens up new diagnostic and therapeutic possibilities for MASLD.

## 1. Introduction

Metabolic dysfunction-associated steatotic liver disease (MASLD) is the most common chronic liver disease [1,2,3]. Its prevalence increases with obesity and metabolic syndrome [4,5,6] and is particularly high in type 2 diabetes [7,8,9,10]. MASLD is a progressive disease that encompasses both steatosis and steatohepatitis, reflecting the transition from simple lipid accumulation in hepatocytes to the development of inflammation and fibrosis. Furthermore, the disease can progress to cirrhosis and is associated with an increased risk of hepatocellular carcinoma [11,12,13,14]. The term “metabolic dysfunction-associated steatotic liver disease” (MASLD) was proposed in 2023 as part of an international, multi-society Delphi consensus under the auspices of leading hepatology, diabetes, and metabolic associations. This new term has replaced the old term “nonalcoholic fatty liver disease” (NAFLD) [12,15,16,17,18] (in this regard, the current review uses the modern MASLD/MASH nomenclature, while the terms NAFLD/NASH are retained only when presenting the results of studies in which patients were selected according to the previous criteria). This central role of the liver is determined in its involvement in nutrient metabolism. The liver coordinates glucose uptake and release through glycogenesis, glycogenolysis, glycolysis, and gluconeogenesis [19]; fatty acid synthesis, oxidation, and esterification; lipoprotein assembly and secretion [19]; amino acid metabolism and plasma protein synthesis [19,20]; and bile acid synthesis from cholesterol [21,22]. It synthesizes 85–90% of circulating proteins, including albumin [19,20]. These processes are regulated by hormonal signals, including insulin, glucagon, estrogen, progesterone, leptin, and glucagon-like peptide-1 (GLP-1) [20] and by meal timing, since melatonin, cortisol, and hepatic circadian genes modulate glucose and lipid metabolism according to the time of day [23]. Therefore, a disruption in one branch of this metabolism does not remain isolated: insulin resistance and hyperinsulinemia simultaneously enhance de novo lipogenesis via the transcription factors sterol regulatory element-binding protein 1c (SREBP-1c) and carbohydrate response element-binding protein (ChREBP), increase gluconeogenesis, alter the secretion of triglyceride-rich very-low-density lipoprotein (VLDL), and contribute to atherogenic dyslipidemia [24,25]. Furthermore, in MASLD, levels of hepatic signaling proteins—hepatokines such as FGF21 and fetuin-A—are altered, which contributes to the systemic nature of the disease and links it to cardiovascular, renal, and oncological risks [25].

In MASLD, the liver is not an isolated target but rather a central link in the vicious cycle of systemic metabolic dysfunction. Hepatic steatosis, insulin resistance, and chronic low-grade inflammation form common pathogenic mechanisms that unite liver, myocardial, and renal pathology, as well as adipose tissue dysfunction, into a single continuum [26,27]. In 2023, the American Heart Association formalized this concept as the cardiovascular–kidney–metabolic (CKM) syndrome [26,28]. Subsequent proposals have expanded this concept to a cardiovascular–kidney–hepatic–metabolic framework, explicitly including the liver in the continuum of mutually reinforcing organ pathologies [26,29,30,31]. The clinical significance of this approach is confirmed by the fact that the fatty liver index (FLI) is an independent predictor of cardiovascular outcomes across the entire spectrum of CKM syndrome [32].

The classical model of MASLD pathogenesis assigned a central role to insulin. Under physiological conditions, insulin suppresses lipolysis in adipose tissue [33,34], inhibits gluconeogenesis in the liver [35,36], and simultaneously stimulates de novo fatty acid synthesis, largely through the activation of the SREBP-1c transcription factor [35,36,37].

According to a widely accepted concept, obesity is associated with the development of selective (dissociated) hepatic insulin resistance. The liver loses its sensitivity to insulin-mediated suppression of glucose production but retains—and, according to some data, even enhances—its lipogenic response to insulin [38,39,40]. However, a recent study has cast doubt on the preservation of direct insulin-mediated lipogenesis in patients with MASLD, revealing a proximal block of insulin signaling at the receptor level and a reduction—rather than an increase—in glucose-stimulated lipogenesis [41]. This uncertainty remains a matter of debate in current reviews, which acknowledge that the question of direct insulin regulation of lipogenesis in MASLD remains open [35,38]. Irrespective of the molecular mechanism, the key metabolic consequence of the described abnormalities remains the simultaneous presence of hyperglycemia and enhanced lipogenesis. This combination of hyperglycemia and excessive lipogenesis lies at the metabolic core of steatosis. Nevertheless, the insulin-centric paradigm does not fully explain the observed phenomena—in particular, the dissociation between the degree of steatosis, the severity of inflammation, and the rate of fibrogenesis in an individual patient.

Over the past decade, the view has emerged that hepatic lipid metabolism is regulated not only by insulin but also by a broad network of non-canonical, insulin-independent signals, including endocrine (glucagon, thyroid hormones, adipokines), paracrine (cytokines, growth factors), and neuronal influences [42,43,44,45,46,47,48]. These mediators are produced by adipose tissue, the intestine, skeletal muscle, and the central nervous system and reach the liver via multiple interorgan axes. The overall architecture of this regulatory network and the interorgan axes that connect it are shown in Figure 1.

The aim of this review is to systematize current data on key canonical and non-canonical regulators of lipid metabolism in the liver and the “liver–other organs” axis, such as the fibroblast growth factors FGF21 and FGF19, adipokines, apolipoprotein C-III, and monoamine neurotransmitters, as well as to demonstrate how an imbalance between non-canonical regulators and canonical mechanisms drives the progression of MASLD and identifies targets for therapeutic intervention.

This is a narrative review. A literature search was performed in the PubMed/MEDLINE and Scopus databases using combinations of the terms “MASLD/NAFLD,” “hepatic lipid metabolism,” “de novo lipogenesis,” “FGF21,” “FGF19,” “adiponectin,” “leptin,” “ApoC-III,” “serotonin,” “dopamine,” “gut–liver axis,” “liver–adipose axis,” “brain–liver axis,” “liver–spleen axis,” and “ceramides,” without being limited to these terms. Priority was given to randomized trials, meta-analyses, and mechanistic studies published in high-impact peer-reviewed journals over the past ten years. Key earlier publications were retained when describing the underlying data. Case reports, conference abstracts, and non-peer-reviewed publications were not considered.

## 2. Canonical Regulators of Hepatic Lipid Metabolism

The canonical regulation of hepatic lipogenesis is mediated by several transcription factors and energy sensors that collectively integrate hormonal (insulin) and nutrient (glucose) signals. Key components of this pathway include the insulin-dependent transcription factor SREBP-1c, the glucose-sensitive ChREBP, and the ubiquitously expressed upstream stimulatory factor 1 (USF-1), which facilitates the recruitment of SREBP-1c to the promoters of lipogenic genes. Insulin also relieves FOXO1-mediated repression. The mechanistic target of rapamycin complex 1 (mTORC1) and AMP-activated protein kinase (AMPK) sensors integrate of energy status, activating and inhibiting lipogenesis, respectively [35,36,49].

SREBP-1c serves as the central regulator of lipogenesis. In response to insulin, the phosphoinositide 3-kinase (PI3K)/protein kinase B (AKT)/mTORC1 cascade stimulates the processing and nuclear translocation of SREBP-1c, which then induces the expression of the full set of fatty acid synthesis enzymes, including ATP-citrate lyase (ACLY), acetyl-CoA carboxylase (ACC), and fatty acid synthase (FASN) [37,49,50,51]. Concurrently, the nutrient sensor ChREBP is activated by the metabolites of glucose and fructose and mediates insulin-independent induction of lipogenic genes [52,53,54,55]. The role of the ChREBP transcription factor is particularly heightened during excessive fructose consumption—a key component of the modern “Western” diet—since fructose, unlike glucose, bypasses the regulatory inhibition of phosphofructokinase and potently activates ChREBP-dependent induction of lipogenic genes [56,57,58,59,60]. Together, SREBP-1c and ChREBP form the transcriptional basis for de novo fatty acid synthesis in the liver [54,61,62]. This process contributes disproportionately to the accumulation of the hepatic triglyceride pool in MASLD. While de novo lipogenesis accounts for about 5% of intrahepatic triglycerides in healthy individuals, this figure rises to approximately 26% in MASLD [63]. Both factors are required for maximal postprandial induction of lipogenic genes [54,61,62].

The opposing, catabolic regulatory pathway is mediated by the nuclear receptors peroxisome proliferator-activated receptor α and δ (PPARα, PPARδ), which activate the transcription of fatty acid β-oxidation genes, and the energy sensor AMPK. By phosphorylating acetyl-CoA carboxylase, AMPK reduces malonyl-CoA levels, which removes the inhibition of carnitine palmitoyltransferase-1 (CPT-1) and switches hepatocytes from lipid synthesis to lipid oxidation [64,65,66]. The liver X receptor (LXR), by contrast, acts as an upstream activator of SREBP-1c and enhances lipogenesis [49,67,68]. The balance between these opposing processes determines whether a hepatocyte will store or oxidize fatty acids. Under physiological conditions, this balance ensures the hepatocyte’s metabolic flexibility, allowing it to rapidly switch from lipogenesis to fatty acid oxidation depending on substrate availability and the body’s energy requirements. In MASLD, however, this balance is disrupted, creating a vicious cycle of progressive steatosis.

The key to understanding steatosis is the phenomenon of selective insulin resistance. Brown and Goldstein formulated the “pathogenic paradox,” building on an earlier hypothesis by McGarry: in obesity, insulin ceases to suppress gluconeogenesis (via FOXO1) but continues to stimulate lipogenesis (via SREBP-1c), resulting in the coexistence of hyperglycemia and hypertriglyceridemia [35,38,69,70]. The molecular basis for this dissociation was elucidated by studies of insulin signaling bifurcation. The mTORC1 pathway is necessary for stimulating lipogenesis but not for suppressing glucose production. Rapamycin at subnanomolar concentrations completely blocks the insulin-induced increase in SREBP-1c mRNA but does not affect the suppression of phosphoenolpyruvate carboxykinase (*PEPCK*) mRNA, which explains the preservation of the lipogenic response despite resistance to the hormone’s glycoregulatory action [71,72].

In addition, regulatory microRNAs constitute another level of control over the insulin cascade by modulating the expression of its key components. For example, miR-424-5p, miR-15b, miR-96, and miR-195 directly suppress insulin receptor (INSR) expression at the mRNA and protein levels; miR-222, miR-126, miR-29a, and miR-135a suppress the insulin receptor substrates IRS1 and/or IRS2; miR-128a, miR-378, and miR-29a reduce the levels of PI3K regulatory subunits, while miR-99a, miR-100, and miR-199a target mTORC1 [73,74,75,76]. Non-canonical regulators, which can both amplify and correct the metabolic imbalance described above, operate against this canonical background. These include post-translational modifications of key transcription factors, alternative signaling cascades, and factors that modulate the stability of SREBP-1c [35,38,77,78,79].

## 3. Non-Canonical Regulators of Lipid Metabolism

### 3.1. Fibroblast Growth Factors: FGF21 and FGF19

Endocrine fibroblast growth factors form a distinct subfamily that lacks a heparin-binding domain and is therefore capable of systemic circulation. FGF19 and FGF21 require the obligate co-receptor β-Klotho (KLB), whereas FGF23 uses α-Klotho. This dependence on Klotho co-receptors confers tissue specificity to their actions [47,80,81,82]. FGF21 and FGF19 act as central non-canonical regulators of hepatic lipid metabolism [47,83]. In particular, FGF21 suppresses de novo lipogenesis, enhances β-oxidation of fatty acids, and improves insulin sensitivity [47,84,85], while FGF19, secreted by enterocytes in response to bile acids, inhibits bile acid synthesis and also promotes lipid oxidation, thereby limiting hepatic steatosis [47,83].

FGF21 is synthesized primarily by hepatocytes in response to metabolic stress—such as fasting, excess carbohydrates, protein deprivation, and lipotoxicity [86,87]. It acts as a metabolic messenger that restores energy homeostasis [86]. FGF21 levels are significantly elevated in patients with obesity and MASLD, reflecting a state of chronic metabolic stress [88].

FGF21 exerts its metabolic effects by binding to the FGFR1c/β-Klotho receptor complex [89,90,91]. In adipose tissue, signaling through this complex increases insulin sensitivity [90,92,93,94,95] and stimulates thermogenesis, thereby increasing total energy expenditure [89,90,91,92,94,95,96,97]. In addition, FGF21 stimulates fatty acid oxidation, primarily in the liver, which helps reduce lipotoxicity [92,94,95]. Concurrently, FGF21 acts on the central nervous system (CNS), specifically on glutamatergic neurons, modulating energy balance and further contributing to energy expenditure and weight loss [91,98]. In an experimental model of nonalcoholic steatohepatitis, FGF21 exerts a direct hepatoprotective effect by reducing lipotoxic damage, suppressing the activation of Kupffer cells and the recruitment of monocyte-derived macrophages, and limiting the development of fibrosis [99].

Importantly, a significant portion of FGF21’s antisteatotic effect is mediated not directly but via the central nervous system [98,100]. A recent study demonstrated that the coordinated action of FGF21 on the brain and liver leads to the regression of metabolic dysfunction-associated steatohepatitis (MASH) [98]. This finding suggests that FGF21 functions at the intersection of the “liver–brain” and “liver–adipose tissue” axes [100,101].

In MASLD and obesity, the concentration of endogenous FGF21 in the blood is consistently elevated, and the degree of this elevation correlates positively with body mass index, hepatic fat content, the severity of insulin resistance, and an adverse lipid profile [102,103,104]. Paradoxically, high levels of the hormone are not accompanied by the expected metabolic improvements. Hyperglycemia, hypertriglyceridemia, and steatosis persist, and endogenous FGF21 is unable to suppress lipolysis in adipocytes, activate β-oxidation of fatty acids in the liver, or increase energy expenditure in brown adipose tissue [102,105]. This has provided a basis for considering obesity and MASLD as states of FGF21 resistance, analogous to the classic phenomena of insulin and leptin resistance [102,106]. The molecular basis of this resistance consists of several parallel mechanisms, such as reduced expression of the β-Klotho co-receptor and the FGFR1c receptor in white and brown adipose tissue, as well as in the liver [102,104], attenuation of FGF21-induced ERK1/2 phosphorylation and suppression of the transcription of immediate-early genes (*Egr1*, *cFos*) in both adipocytes and hepatocytes [102], as well as chronic low-grade inflammation and oxidative stress, which further impair signal transduction [103,105]. However, FGF21 resistance is not absolute. Pharmacological doses of exogenous FGF21 or its analogs can overcome this resistance and restore the hormone’s metabolic activity [85,87,107], which explains the significant therapeutic potential of FGF21 mimetics in MASLD and steatohepatitis [87,102,104,108]. Thus, dysfunction of the FGF21 axis is both a marker of metabolic dysfunction and a promising therapeutic target, while the concept of FGF21 resistance complements the insulin-centric model of MASLD pathogenesis, providing a more comprehensive understanding of the interorgan disruption of lipid metabolism.

FGF19, whose murine ortholog is FGF15, is a postprandial intestinal hormone synthesized by ileal enterocytes in response to bile acid absorption and subsequent activation of the FXR [109,110,111]. After entering the liver via the portal vein and binding to the FGFR4/β-Klotho complex, FGF19 inhibits CYP7A1, a key enzyme in bile acid synthesis, thus completing the negative feedback loop of the enterohepatic circulation [110,111,112,113].

In addition to regulating the bile acid pool, FGF19 suppresses hepatic lipogenesis and gluconeogenesis independently of insulin and stimulates glycogen and protein synthesis [114,115]. However, FGF19 should be viewed not simply as an insulin antagonist, but as an insulin-independent postprandial regulator that coordinates bile acid synthesis, glycogen and protein metabolism, lipogenesis, and gluconeogenesis, partially mimicking certain postprandial effects of insulin without fully duplicating classical insulin signaling [114,115,116]. Disruptions in the “FXR–FGF19/FGF15” axis, driven by dysbiosis and changes in the bile acid profile, are associated with steatosis and steatohepatitis [117,118], whereas restoration of this signaling attenuates MASLD in experimental models [117]. Thus, FGF19 serves as a molecular hub of the “gut–liver” axis.

### 3.2. Adipokines: Adiponectin and Leptin

Adipose tissue functions as a bona fide endocrine organ that secretes adipokines, which convey information about energy stores to the liver [119,120,121]. Adiponectin and leptin represent two opposing aspects of this communication.

Adiponectin is the most abundant adipokine in the bloodstream. Unlike most other adipokines, its concentration decreases in cases of obesity and insulin resistance [122,123,124,125,126,127]. The binding of adiponectin to its receptors AdipoR1 and AdipoR2 leads to the activation of AMPK and the ceramide-hydrolyzing activity inherent in these receptors [128,129]. By hydrolyzing proapoptotic, insulin-desensitizing ceramides to sphingosine, which can subsequently be phosphorylated to sphingosine-1-phosphate, this activity helps restore insulin sensitivity and reduce hepatic steatosis [128,129]. The adiponectin-AMPK axis suppresses hepatic lipogenesis, enhances fatty acid oxidation, and exerts anti-inflammatory and antifibrotic effects [130,131]. The activity of the adiponectin-AMPK axis extends to the kidney, linking adiponectin to the renometabolic component of the CKM syndrome [132,133]. Hypoadiponectinemia is considered one of the predictors of the progression of steatosis to steatohepatitis [134,135,136,137].

While adiponectin acts primarily as a hepatoprotective adipokine, a deficiency of which contributes to the progression of steatosis to steatohepatitis, the role of leptin is significantly more complex. Leptin exhibits a complex, context-dependent role. Under physiological conditions, leptin acts on the dorsal vagal complex (the center of parasympathetic regulation in the brainstem) and the hypothalamus. This central activation reduces lipid content in the liver. It increases the secretion of triglycerides in VLDL by approximately 30%. At the same time, it suppresses de novo lipogenesis. This latter effect is based on the inhibition of key lipogenic enzymes—FASN and acetyl-CoA carboxylase (ACC) [138,139,140,141]. This antisteatotic effect is independent of reduced food intake or body weight and is mediated via the vagus nerve [141]. In contrast, systemic administration of an equivalent dose of leptin does not affect hepatic lipid content, confirming an exclusively central mechanism of action [138]. In obesity, selective leptin resistance develops, and the hormone’s protective central effects—including stimulation of triglyceride secretion and suppression of de novo lipogenesis—are lost [142,143]. Pharmacological restoration of leptin sensitivity is possible, in particular, by inhibiting the histone deacetylase HDAC6. In diet-induced obese mice, the selective HDAC6 inhibitor tubastatin A reduces food intake by approximately 25–30%, halves fat mass, significantly alleviates hepatic steatosis, and improves systemic glucose homeostasis. These effects are absent in models with defective leptin signaling (db/db, ob/ob, *MC4R*-knockout), confirming a leptin-dependent mechanism of action [144].

In peripheral tissues, by contrast, chronic hyperleptinemia becomes profibrogenic. Leptin directly stimulates the production of type I collagen in activated hepatic stellate cells (HSCs) through the Janus kinase/signal transducer and activator of transcription (JAK/STAT) pathway [145], and its deficiency in rodents completely prevents the development of fibrosis in response to chronic liver injury [146]. In contrast to rodents, leptin deficiency in large animals reproduces the full spectrum of MASLD, progressing to advanced fibrosis (as demonstrated in leptin-deficient (*Leptin*^−/−^) pigs, in which fibrosis develops in 43% of cases by 22–35 months) [147]. Activation of AMPK-dependent pathways suppresses the profibrogenic effects of leptin in HSCs: adiponectin, by activating AMPK, inhibits leptin signaling via the JAK2/STAT3 pathway, reducing tissue inhibitor of metalloproteinases-1 (TIMP-1) expression and enhancing suppressor of cytokine signaling 3 (SOCS3) production [148,149]. In turn, leptin deficiency is accompanied by suppressed AMPK phosphorylation in the liver, which triggers mitophagy, enhances oxidative stress, and promotes fibrogenesis [147].

It is important to emphasize that leptin’s profibrogenic potential is context-dependent. It is determined by energy status, the degree of obesity and leptin resistance, the severity of inflammation, the condition of adipose tissue, sex differences, and the specific disease model. For example, in the liver, leptin acts as a profibrogenic factor [145,146]. In contrast, in white adipose tissue, leptin suppresses fibrosis by reducing the expression of the integrin ITGA5 and attenuating PI3K–AKT signaling [150]. Furthermore, under conditions of leptin deficiency, other adipokines take over the profibrogenic function, particularly lipocalin-2, which directly activates HSCs and exacerbates steatohepatitis [151]. These opposing directions of effect caution against an oversimplified, one-size-fits-all interpretation of leptin’s role in the pathogenesis of fibrosis.

### 3.3. Apolipoprotein C-III

Apolipoprotein C-III (ApoC-III) is a small (79 amino acids) exchangeable apolipoprotein synthesized primarily by hepatocytes and, to a lesser extent, by enterocytes [152,153,154]. In the bloodstream, it acts as a potent inhibitor of lipoprotein lipase (LPL), suppressing the hydrolysis of triglycerides in chylomicrons and VLDL [153,155]. At the same time, ApoC-III impedes the hepatic uptake of triglyceride-rich lipoproteins by disrupting the interaction of apolipoprotein E with the LDLR and LRP1 receptors, which collectively significantly slows the clearance of atherogenic particles from the bloodstream [152,153,154,156].

In addition to its effect on lipoprotein catabolism, ApoC-III directly stimulates the intrahepatic assembly and secretion of triglyceride-rich VLDL, promoting the recruitment of additional triglycerides to the nascent particles [157,158]. Thus, ApoC-III directly links the intrahepatic triglyceride pool to systemic hypertriglyceridemia. Elevated ApoC-III expression is associated with atherogenic dyslipidemia, high residual cardiovascular risk [153,154,157], and severe forms of hypertriglyceridemia, including familial chylomicronemia syndrome (FCS) and multifactorial chylomicronemic syndrome (MCS) [159,160].

In the context of the “liver–vasculature” axis, ApoC-III acts as a central mediator linking the atherogenic dyslipidemia observed in hepatic steatosis to the cardiovascular component of metabolic syndrome [161]. Elevated ApoC-III expression, characteristic of insulin resistance and steatosis, contributes to hypertriglyceridemia by inhibiting lipoprotein lipase-mediated triglyceride hydrolysis and hepatic clearance of remnant particles [161]. In contrast, genetic inactivation of *APOC3* leads to lifelong low concentrations not only of triglycerides but also of atherogenic cholesterol in remnant lipoproteins [162]. In a large population-based study, heterozygous carriage of loss-of-function *APOC3* mutations was associated with a 41% reduction in the risk of ischemic vascular disease (hazard ratio (HR) 0.59; 95% confidence interval (CI) 0.41–0.86) and a 36% reduction in the risk of ischemic heart disease (HR 0.64; 95% CI 0.41–0.99) [163]. This cardioprotective effect is mediated primarily by low levels of remnant cholesterol, rather than low-density lipoprotein cholesterol [162]. The antisense oligonucleotide olezarsen and the small interfering RNA (siRNA) plozasiran, which inhibit hepatic synthesis of ApoC-III, have already demonstrated in randomized, placebo-controlled trials their ability to significantly reduce triglyceride levels and the incidence of acute pancreatitis episodes in patients with FCS [164].

It should be emphasized that, unlike resmetirom, the first drug approved specifically for the treatment of MASH [165,166,167], and semaglutide, which has demonstrated the ability to resolve MASH in clinical trials [168], ApoC-III is not a direct therapeutic target for the treatment of MASLD/MASH. Despite its close functional link to hepatic lipid metabolism, ApoC-III, as currently understood, is not the primary determinant of the development of hepatic steatosis [161]. Its clinical significance for MASLD is primarily indirect and manifests through its contribution to the development of atherogenic dyslipidemia, the accumulation of remnant lipoproteins in plasma, and the resulting increased cardiovascular risk [162,163,169]. Accordingly, drugs that inhibit ApoC-III (olezarsen, plozasiran, volanesorsen) occupy a fundamentally different clinical niche and are intended for the treatment of severe hypertriglyceridemia and the prevention of acute pancreatitis in FCS [164,170].

### 3.4. Neurotransmitters: Serotonin and Dopamine

Monoamine neurotransmitters, traditionally associated with the central nervous system, have a pronounced peripheral effect on metabolism. The vast majority of serotonin in the body is synthesized not in the brain but in enterochromaffin cells of the intestine [171,172]. As a result, serotonin acts as a natural humoral mediator of the “gut–liver” axis. Serotonin produced in the intestine enters the portal circulation and directly acts on hepatocytes via the 5-hydroxytryptamine receptor 2A (HTR2A), regulating de novo lipogenesis and contributing to the development of hepatic steatosis [173]. A study demonstrating that gut-derived serotonin directly regulates hepatic steatosis was pivotal in identifying this axis. It was found that a local increase in serotonin concentration in the portal blood following a high-fat diet activates the hepatic HTR2A receptor. Tissue-specific knockout of tryptophan hydroxylase-1 (TPH1) in the intestine or the HTR2A receptor in the liver renders animals resistant to steatosis without affecting systemic energy metabolism, and a selective HTR2A antagonist prevents fat accumulation in the liver [173].

The original study of the gut TPH1–liver HTR2A axis and its role in the development of steatosis [173] was extended by subsequent studies, which directly linked it to the progression of steatohepatitis. In particular, activation of HTR2A in hepatocytes triggers lipogenesis and an inflammatory response via the HTR2A/PPARγ2 signaling pathway [174], which represents one of the key mechanisms through which gut serotonin contributes to the transition from simple steatosis to steatohepatitis. In addition, gut-derived serotonin, acting through the same HTR2A receptor, directly induces endoplasmic reticulum (ER) stress in hepatocytes [175], adding yet another independent mechanism of hepatocellular damage and disease progression. The effects described occur within the context of a more complex regulatory network. Serotonin signaling is closely integrated with the gut microbiota, which regulates the synthesis, intracellular storage, reuptake, and catabolism of this neurotransmitter [176,177,178], embedding it within a broader microbe-host communication network. From a therapeutic standpoint, peripheral targeting of HTR2A represents a promising preclinical strategy, potentially enabling an antisteatotic effect with minimal central side effects [179,180]. However, its clinical efficacy and safety in MASLD have not yet been confirmed in controlled trials [180]. Similar principles of peripheral neurotransmitter control apply to another monoamine—dopamine.

Dopamine acts beyond the nervous system. Peripheral dopamine directly affects insulin-sensitive tissues—the liver, adipose tissue, and skeletal muscle—by modulating insulin signaling and metabolic functions [181]. In the liver, it stimulates glucose uptake via D2R, independent of the insulin receptor. In skeletal muscle, it enhances glucose uptake and activates AMPK via D1R, and in white adipose tissue, it potentiates insulin-mediated glucose uptake and regulates lipid catabolism [181]. Clinical evidence supporting the importance of dopaminergic regulation comes from bromocriptine—a D2R agonist approved for the treatment of type 2 diabetes. It also remodels dopamine signaling in adipose tissue by increasing the expression of D1R and tyrosine hydroxylase (TH), and activates catabolic pathways, thus improving the metabolic profile [182].

In models of metabolic syndrome, dopamine receptor agonism—specifically, chronotherapy with bromocriptine—corrects neuroendocrine and metabolic abnormalities by normalizing the monoaminergic tone of the ventromedial hypothalamus and simultaneously reducing the expression of proinflammatory (NF-κB, JNK, SOCS-3), lipogenic (SREBP1, mTORC1), and gluconeogenic (PEPCK, G6Pase) factors in the liver [183]. These data underscore the key role of the “brain–liver” axis in regulating glycemia and lipid metabolism.

However, unlike better-studied endocrine factors such as FGF21, FGF19, and adipokines, the direct effect of dopamine on hepatic lipid metabolism has been described primarily in experimental studies, and its clinical significance requires further investigation [184]. Indeed, although dopamine receptors are expressed in the liver and are involved in the modulation of insulin signaling and gluconeogenesis [185], there are insufficient systematic clinical studies on their role in steatosis in MASLD. Preclinical data, including recent studies with levodopa that have demonstrated its ability to attenuate liver fibrosis via D1R/Hippo/YAP signaling [186], as well as broader studies on catecholamines [185], point to the promising hepatoprotective effects of the dopaminergic system. However, their translation into clinical practice remains a distant prospect, as the mechanisms of action have not yet been fully elucidated, and systematic clinical trials have not yet been conducted [185,186].

Thus, serotonin and dopamine are two evolutionarily ancient but as yet insufficiently studied neurotransmitters that, via HTR2A receptors and dopamine receptors, respectively, participate in the regulation of hepatic lipid metabolism within the “gut–liver” and “brain–liver” axes. A summary of all the non-canonical regulators discussed in this section, their key receptor targets, interorgan axes, and the direction of their effects on hepatic lipid metabolism is presented in Table 1.

### 3.5. The Role of Ceramides as Signaling Molecules in MASLD

The ceramidase activity of adiponectin receptors represents only one component of a broader sphingolipid regulatory network that links hepatocyte lipid load to insulin sensitivity. What matters is not total ceramide content but ceramide structure (acyl chain length, presence of a 4,5-trans double bond) and site of synthesis—different ceramides exert opposite effects in different cell types. De novo ceramide synthesis begins with serine palmitoyltransferase (SPT). Induction of its SPTLC2 subunit (serine palmitoyltransferase long-chain base subunit 2) during endoplasmic reticulum stress and liver-specific overexpression of this gene in mice increased ceramide levels, raised fasting blood glucose, and reduced insulin receptor phosphorylation [187]. The final stage of synthesis is no less important. The enzyme dihydroceramide desaturase 1 (DES1), encoded by the *Degs1* gene in mice (the ortholog of *DEGS1* in humans), incorporates a 4,5-trans double bond into the sphingoid base of ceramides [188]. Inducible or tissue-specific deletion of *Degs1* in the liver and/or adipose tissue eliminated steatosis and insulin resistance in obese mice [188]. However, dihydroceramides lacking this double bond did not reproduce the effects of ceramides: they did not inhibit Akt/PKB, did not induce lipogenic genes, and did not inhibit hormone-sensitive lipase [188].

This selectivity also extends to the length of the acyl chain. Deletion of *CerS6*, but not *CerS5*, protected animals from obesity and insulin resistance, and the C16:0 sphingolipids formed by CerS6 interacted with the mitochondrial fission factor Mff and promoted mitochondrial fragmentation [189]. Deletion of *CerS6* or *Mff* protected against fatty acid-induced mitochondrial fragmentation and improved glucose tolerance and insulin sensitivity in obese mice [189]. In *CerS2* haploinsufficiency, compensatory accumulation of C16:0 ceramides suppressed β-oxidation due to inactivation of electron transport chain components and increased susceptibility to steatohepatitis and insulin resistance [190]. In a model of glucocorticoid-induced insulin resistance, ceramides activate PP2A and PKCζ, leading to the inhibition of Akt [191]. However, this is not the only pathway: the suppression of ceramide synthesis by myriocin and the antisense oligonucleotide (ASO)-mediated reduction in *Degs1*/*DEGS1* (DES1) improved hepatic insulin sensitivity independently of the reduction in C16:0 ceramides—by reducing plasma membrane sn-1,2-diacylglycerol (PM sn-1,2-DAG) levels and protein kinase C epsilon (PKCε) activity, which reduced phosphorylation of the insulin receptor at threonine 1150 (INSR Thr1150) and restored insulin-stimulated phosphorylation of the insulin receptor at tyrosine 1152 (INSR Tyr1152) and Akt at serine 473 (Akt Ser473) [192].

A 2025 study [193] demonstrated the link between sphingolipid metabolism and the “gut-liver” axis. The symbiotic fungus *Fusarium foetens* induced regression of established steatohepatitis in mice through an intestinal ceramide synthase 6 (CerS6)-ceramide axis, and its secondary metabolite FF-C1 acted as a CerS6 inhibitor [193].

Clinical data outline the limits of the diagnostic value of these lipids. In patients who underwent liver biopsy, the total content of hepatic ceramides in steatohepatitis exceeded that in simple steatosis by 50%, while dihydroceramides (16:0, 22:0, 24:1) were elevated only in steatohepatitis [194]. At the same time, in a sample of 181 patients who underwent surgery for obesity, ceramides, dihydroceramides, and hexosylceramides increased in parallel with steatosis, but did not differ between simple steatosis and steatohepatitis, although the addition of lipid markers improved the accuracy of steatohepatitis diagnosis, with the area under the receiver operating characteristic curve (AUROC) increasing from 0.667 to 0.785 (*p* = 0.025) [195]. In a population-based study (*n* = 1131, median follow-up 13.3 years), ceramide ratios (Cer(16:0)/Cer(24:0), Cer(18:0)/Cer(24:0), Cer(24:1)/Cer(24:0)) predicted major adverse cardiovascular events (MACE) better than absolute concentrations [196]. The fibrogenic component of sphingolipid regulation is partially autonomous: sphingosine 1-phosphate (S1P) is elevated in the liver in steatohepatitis and activates stellate cells, while blockade of the sphingosine-1-phosphate receptor 2 (S1PR2) or knockout of the *S1pr2* gene reduced fibrosis without affecting the severity of steatosis or hepatocyte damage [197]. Data on the effects of therapy are still limited: in type 2 diabetes, liraglutide (1.2 mg/day for 6 months) reduced total plasma dihydroceramide (DhCer) levels by 15.1% (*p* = 0.005), and this reduction was independently associated with a decrease in liver fat content [198].

## 4. Interorgan Regulatory Axes of Hepatic Lipid Metabolism

In Section 3, non-canonical regulators are considered separately—as molecules with a specific receptor and an intracellular signaling cascade. In this section, the same mediators are analyzed as components of interorgan connections; therefore, the focus shifts to the source of the metabolite, its route to the liver, and the outcome of the simultaneous integration of multiple inputs. The molecular mechanisms are not repeated here; instead, cross-references to the relevant subsections are provided.

### 4.1. The “Liver–Adipose Tissue” Axis

Adipose tissue and the liver form a functional metabolic unit, in which signal exchange—primarily via the flow of free fatty acids and adipokines—largely determines the fate of lipids in the body [161]. In insulin resistance and adipocyte dysfunction, the antilipolytic action of insulin is disrupted, resulting in unregulated lipolysis that releases an excess of non-esterified fatty acids, which—especially from visceral adipose tissue—enter the liver directly via the portal system [199,200]. It is precisely the insulin resistance of adipose tissue—and not just that of the liver—that determines the influx of substrate for triglyceride synthesis. The marker of hepatic macrophage activation, sCD163, correlates significantly with indices of adipocyte insulin resistance, but not with hepatic insulin resistance [201]. This indicates a close link between adipose tissue dysfunction and inflammation in the liver in steatohepatitis.

Acute activation of adipocyte lipolysis rapidly reprograms the entire hepatic lipidome, indicating a dynamic coupling between the two organs [202]. On a high-fat diet, excessive activation of autophagy is observed in white adipose tissue. This exacerbates the pathological interaction between adipose tissue and the liver; specifically, it enhances lipolysis, increases the influx of free fatty acids to the liver, and thereby accelerates the development of steatosis [203]. The adipokines adiponectin and leptin, whose molecular mechanisms of action are discussed in Section 3.2, serve as humoral mediators of the “liver–adipose tissue” axis [119,120,122,128,129,138,141], and the nature of the liver’s response to lipolysis depends on the body’s nutritional status [204]. Thus, adipose tissue functions not only as a lipid store but also as a central endocrine regulator of hepatic metabolism, and its dysfunction triggers a cascade of events leading to steatosis and inflammation. Similar principles of humoral and metabolic communication are also at work in another key axis—the “gut–liver” axis.

### 4.2. The “Gut–Liver” Axis

Anatomically, the liver receives about 70% of its blood supply from the portal vein and is therefore the first organ to encounter substances of intestinal origin—microbiota metabolites, bile acids, bacterial endotoxins, and neurotransmitters. Impaired intestinal barrier function and dysbiosis trigger portal endotoxemia and inflammation. Molecular bacterial products—primarily lipopolysaccharide—penetrate into the portal circulation through the damaged intestinal wall, activating Toll-like receptor 4 (TLR4)-dependent proinflammatory cascades in the liver [205,206]. This sequence of events forms the pathophysiological basis for both the progression of steatosis and fibrosis and for targeted therapeutic interventions. Bile acids serve as the central mediators of the “gut–liver” axis. Through the nuclear FXR and the membrane receptor TGR5, they regulate FGF19 synthesis, lipogenesis, and inflammation [112,207]. In enterocytes, FXR activation induces the secretion of FGF19, which reaches the liver via the portal circulation and suppresses the expression of lipogenesis-related genes, as well as limits NF-κB-dependent inflammation. Concurrently, secondary bile acids stimulate the release of GLP-1 via TGR5 and exert an independent anti-inflammatory effect [207].

Modulation of the microbial bile acid profile determines the direction of signaling. For example, hyodeoxycholic acid attenuates steatosis through the “gut–liver” axis. By inhibiting intestinal FXR, it activates an alternative pathway for bile acid synthesis (CYP7B1) and promotes the enrichment of *Parabacteroides distasonis* in the microbiota, which—through the resulting γ-linolenic acid and the activation of PPARα in the liver—leads to the suppression of lipogenesis [208]. Similarly, enrichment of the microbiota with *Akkermansia muciniphila* improves the course of MASLD by correcting the bile acid profile, specifically by reducing the pool of secondary bile acids and activating the intestinal FXR–FGF15 axis [209,210]. In addition to microbial metabolites, intestinal serotonin (Section 3.4) and postprandial FGF19 (Section 3.1) act along this same axis, making it an integrative hub of non-canonical regulation.

The range of microbial signals reaching the liver is not limited to bile acids and serotonin [211]. Short-chain fatty acids, such as acetate, butyrate, and propionate, have been studied most extensively. Butyrate suppresses lipogenic and activates oxidative transcriptional programs in hepatocytes via activation of the CaMKII–CREB pathway and inhibition of the HDAC1–CREB pathway, and this effect is lost in mice with knocked-out FFAR3 (GPR41) and FFAR2 (GPR43) receptors [212]. In particular, butyrate reduces the expression of *Acc1*, *Fasn*, *Scd1*, and *PPARγ* and increases the expression of CPT1a, *Cyp4a10*, *Cyp4a14*, and *Mcad* [212]. Increased intestinal propionate production alleviates steatosis by activating the “adiponectin (APN)–AMPK–PPARα” axis, which is accompanied by the inhibition of *ACC*, *FAS*, *SREBP-1c*, and *SCD1*, and the activation of *AMPK*, *PPARα*, and *ACOX1* [213]. Acetate exhibits dual effects. On the one hand, it is essential for the protective action of prebiotic fibers. Knockout of the *Ffar2* gene completely abolishes the reduction in steatosis and fibrosis [214]. On the other hand, it serves as a substrate for de novo fatty acid synthesis via acetyl-CoA synthetase 2 (ACSS2). In a clinical study, 42 days of inulin supplementation (20 g/day) in patients with fatty liver disease was accompanied by an increase in intrahepatocyte lipid content (IHCL) from 20.9% to 26.8% (*p* = 0.012), whereas supplementation with its propionate ester was not [215]. Dual inhibition of ACLY and ACSS2 by EVT0185 reduced steatosis, inflammation, and fibrosis in MASH models in mice and suppressed de novo lipogenesis in human liver slices [216]. Clinical data do not support a universal benefit of this class of metabolites. In a randomized trial involving 181 patients with MASLD (177 completed treatment), butyrate salts did not alter the severity of steatosis as measured by the Controlled Attenuation Parameter (CAP) (ΔCAP was 0.84 for sodium butyrate versus −0.23 for calcium butyrate (*p* = 0.70)) [217]. However, the sodium salt was associated with a reduction in trimethylamine N-oxide (TMAO) concentrations and a more pronounced decrease in the fatty liver index (FLI) compared with the calcium salt [217]. Furthermore, in lean MASLD, levels of propionate and isobutyrate are elevated, and in 3D cultures of primary human hepatocytes, these metabolites induced lipid accumulation [218]. Thus, the direction of the effect is determined by the site of metabolite formation, the receptor involved, and the metabolite’s subsequent metabolic fate.

Another biologically active group of substances consists of microbial derivatives of tryptophan. Patients with MASLD show reduced levels of indole-3-propionic acid (IPA) and indole-3-acetic acid (IAA) in feces, and administration of these compounds to mice on a Western diet reduces steatosis and inflammation by limiting endotoxin exposure and inactivating macrophages [219]. Indole-3-acetate reduces alanine aminotransferase (ALT) activity, triglyceride levels, hepatocyte ballooning, and lobular inflammation without altering the composition of the microbiota, acting in hepatocytes via the aryl hydrocarbon receptor (AhR) and in macrophages via AMPK [220]. The clinical significance of this pathway is supported by a study of 233 patients with histologically confirmed liver damage: IPA concentrations were lower in patients with fibrosis (*p* = 0.039) and correlated with transcripts associated with stellate cell activation. IPA itself inhibited stellate cell activation in vitro [221]. Circulating indole is also reduced in obesity and alleviates steatosis in mice through PFKFB3 in myeloid cells [222]. Thus, a deficiency of indole metabolites is not a concomitant feature but a potentially modifiable link connecting microbial tryptophan catabolism with fibrogenesis. Trimethylamine N-oxide (TMAO), which is formed during the hepatic oxidation of trimethylamine (TMA) by flavin-containing monooxygenase 3 (FMO3), has the opposite effect [223]. According to a meta-analysis of seven studies (7583 participants), circulating TMAO levels were higher in patients with NAFLD (standardized mean difference, 0.66); however, the very high heterogeneity (I^2^ = 94%) and evidence of publication bias limit the reliability of this estimate [224]. In the prospective PREVEND study (n = 5292; median follow-up 8.2 years), the level of this metabolite was associated with the fatty liver index (FLI) and predicted all-cause mortality only in the NAFLD subgroup—for the top tertile of TMAO compared with the bottom tertile, the adjusted hazard ratio was 1.90 (95% CI 1.18–3.04), but not outside of it [225]. Mechanistically, TMAO shifts the bile acid profile toward FXR antagonists and enhances hepatic lipogenesis, whereas inhibition of CYP7A1 or activation of FXR abolishes this effect [226]. Knocking out *FMO3* in mice attenuates steatosis [223]. At the same time, the concentration of this metabolite reflects both dietary patterns and kidney function; therefore, its causal role in the progression of MASLD in humans remains unproven.

Thus, microbial metabolites are integrated into the regulatory network under consideration through the provision of substrates for lipogenesis (acetate—ACSS2) and through receptor signaling (FFAR2/FFAR3, AhR, FXR). In this context, the “gut-liver” axis, which integrates a wide range of signals (from microbial metabolites to neurotransmitters and hormones), can be viewed as a key integrator of metabolic information originating from the gastrointestinal tract.

### 4.3. The “Brain–Liver” Axis

The central nervous system controls hepatic metabolism through autonomic innervation and neuroendocrine signals. The hypothalamus integrates hormonal (insulin, leptin, ghrelin) and nutrient (glucose, fatty acids) stimuli and, via the sympathetic and parasympathetic divisions of the autonomic nervous system, regulates glucose production and lipid metabolism in the liver [138,227,228]. Sympathetic fibers directly innervate the liver parenchyma and, by releasing norepinephrine, stimulate gluconeogenesis and VLDL production, whereas parasympathetic signals mediated by the vagus nerve play an important role in postprandial regulation of glucose homeostasis and insulin sensitivity [228,229]. Experiments have shown that the development of steatosis depends on the presence of hepatic parasympathetic cholinergic neurons. Their absence alters the liver’s susceptibility to a high-fat diet [230]. It is through this “brain–liver” axis that leptin’s central anti-steatotic effect (Section 3.2) is mediated—specifically, via vagal stimulation of triglyceride export [138,141]—as well as the CNS-mediated metabolic effects of FGF21 (Section 3.1) [98]. The dopaminergic component of this axis is described in Section 3.4: normalization of the circadian peak of dopaminergic activity in the suprachiasmatic nucleus by quick-release bromocriptine (bromocriptine-QR) corrects sympathetic tone and the hypothalamic–pituitary–adrenal (HPA) axis, improving whole-body post-receptor insulin sensitivity [231], while the remodeling of the local dopaminergic system in the liver and adipose tissue complements this central mechanism through a direct peripheral action [182]. It is important to note that both components operate along the same axis but at different points of signal transmission. Along with monoamine neurotransmitters, other signaling molecules originating in the gut also make a significant contribution to the afferent component of the “brain–liver” axis. In particular, bile acids are key bioactive signaling molecules in interoceptive gut–brain communication [232]. They act as humoral mediators that convert changes in the luminal environment (the composition and diversity of the bile acid pool) into a cascade of neurohumoral signals, thereby closing the loop between the three main pathways: endocrine, neuronal, and immune.

As shown in the study by Ní Dhonnabháín et al. [233], activation of nuclear (FXR) and membrane (TGR5) bile acid receptors on enteroendocrine L-cells stimulates the secretion of GLP-1 and peptide YY (PYY) (endocrine pathway). Activation of TGR5 at afferent terminals of the vagus nerve and in enterochromaffin cells initiates direct neuronal transmission to the CNS (neuronal pathway). Finally, modulation of macrophage and dendritic cell functions via the same receptors influences the profile of circulating cytokines, representing an immune-mediated pathway of gut–brain signaling [232]. Together, these findings delineate a neuronal level of control that complements humoral regulation of the liver and completes the ascending signaling pathway within the bidirectional “gut–brain–liver” axis.

### 4.4. The “Liver–Spleen” Axis

The “liver–spleen” axis complements the endocrine and neuronal regulation of hepatic metabolism with an immune component [234]. Blood outflow from the spleen into the portal system provides the anatomical basis for the entry of its cells and soluble mediators into the liver [235]. In obesity, changes in the spleen’s immune composition accompany inflammation of adipose tissue and the liver; therefore, this axis should be considered even outside the context of cirrhosis and its hemodynamic complications [234,235].

A large population-based study of 37,066 participants from the UK Biobank showed that spleen volume is positively and independently associated with the percentage of fat in the liver, liver volume, and the fibrosis-4 (FIB-4) index. An increase in spleen volume was observed even at low levels of hepatic fat (5–20%), indicating spleen involvement at an early stage of the disease [236]. These findings are consistent with the results of an ultrasound study that revealed a positive correlation between portal vein diameter and splenic vein diameter in patients with MASLD [237]. A 2025 study demonstrates the cellular specificity of this association: in obese leptin-deficient BTBR mice, a positive correlation was found between the abundance of natural killer T (NKT) cells and cells with a myeloid-derived suppressor cell (MDSC) phenotype in the spleen and liver, with the correlation being strong for MDSCs and weak for NKT cells [235]. However, the reduction in hepatic T and B lymphocytes was not replicated in the spleen [235]. Consequently, the immune changes in the two organs do not simply reflect a single systemic response. At the same time, a comparison of cellular composition alone does not establish the direction of migration, the suppressive function, or the causal contribution of these populations to steatosis. An additional limitation is the distinctive immune phenotype of leptin deficiency, although the authors confirmed the main results in a model of diet-induced obesity [235]. In clinical interpretation, it is necessary to distinguish between organ size and its immune activity. In patients with histologically confirmed fatty liver disease, spleen size did not correlate with the stage of the disease but was associated with body weight, height, and high-density lipoprotein (HDL) levels [238]. Therefore, splenomegaly alone does not establish either the mechanism of interorgan interaction or its causal role in early-stage disease.

Experiments with interleukin-10 (IL-10) support a causal relationship between splenic regulation and lipid metabolism. In mice on a high-fat diet, splenectomy reduced circulating IL-10, exacerbated triglyceride accumulation and hepatic macrophage infiltration, and worsened glucose intolerance [239]. Steatosis increased despite reduced food intake and body weight, whereas in animals with an intact spleen under comparable dietary restriction, it decreased. Administration of IL-10 attenuated these abnormalities, and in *Il10* knockout mice, the additional effect of splenectomy on tissue inflammation was minimal [239]. These data highlight the loss of anti-inflammatory protection as a mechanism that cannot be reduced to excess calories. At the same time, the systemic effects of splenectomy and cytokine administration do not allow for a complete separation of the direct effects on the liver from changes in adipose tissue and overall metabolism [239]. Experiments on Sprague–Dawley rats [240] provide independent confirmation of the spleen’s role in regulating hepatic lipid metabolism. Splenectomy significantly accelerated the development of hepatic steatosis on a high-fat diet. On a normal diet, no signs of steatosis were detected after splenectomy. The molecular mechanism underlying this effect, observed in animals on a high-fat diet, included a marked decrease in *PTEN* gene expression and PTEN protein levels in the liver, along with a concomitant increase in the pAkt/Akt ratio, indicating activation of the PI3K/AKT signaling pathway associated with lipogenesis. In rats on a normal diet, splenectomy did not cause changes in Akt activity. Splenectomy also increased serum lipid levels (with the exception of triglycerides and high-density lipoproteins) on both a high-fat and a normal diet, which is consistent with the systemic nature of metabolic disturbances [240]. Thus, in addition to the loss of IL-10-dependent anti-inflammatory protection, the spleen may modulate the liver’s susceptibility to steatosis by regulating PTEN/AKT signaling—a mechanism independent of the immune component and deserving of further study in the context of early MASLD. One limitation is that these data were obtained in rodents and require clinical validation.

The deleterious effect may be mediated through immune cell trafficking and soluble signaling molecules. In an experiment involving the photoconversion of spleen cells and spleen transplantation, the migration of monocytes into the fibrosing liver was observed. The CD11b+CD43hiLy6Clo (sM-1) subpopulation, after transformation into sM-2/sMφ, acquired macrophage properties that enhanced the activation of stellate cells, and the medium conditioned by hepatocytes from a fibrotic liver promoted its further reprogramming [241]. Consequently, the outcome of interorgan transfer depends simultaneously on the origin of the immune cell and the state of the recipient tissue [241]. This supports the concept of functional organ interaction but does not yet prove the existence of a closed bidirectional signaling loop in early MASLD. Data from fibrosis models also cannot be automatically extrapolated to the stage of isolated steatosis in humans [234].

A new molecular mechanism has been described for splenic CD8^+^ T cells with high *TRNP1* expression. In mouse models of MASLD/MASH, chromatin remodeling initiated *TRNP1* expression, which in turn increased the expression of the *FURIN* and *CTSD* genes [242]. These proteases, FURIN and CTSD, facilitated the maturation of the insulin receptor (INSR) and the release of its soluble α-subunit (INSR-α), which activates the ERK–p90RSK pathway in hepatic stellate cells [242]. Genetic knockout of the *Trnp1* gene in CD8^+^ T cells reduced fibrosis without significantly altering hepatic lipid accumulation, while neutralizing antibodies against INSR/INSR-α attenuated experimental fibrosis [242]. Corresponding changes in the spleen and CD8^+^ T cells have been observed in patients with MASLD [242]. Here, a component of the canonical insulin system plays a different, interorgan profibrogenic role. A limitation remains that the mechanistic data were obtained primarily in models with choline and/or methionine deficiency (MCDHFD, CDAHFD), whereas TRNP1 induction was less pronounced in high-fat diet (HFD)/Western diet (WD), making it difficult to extrapolate the results to the prevalent obesity-associated MASLD [242].

Thus, it can be hypothesized that the spleen plays a role in regulating the liver’s susceptibility to metabolic stress by balancing anti-inflammatory defense, cell recruitment, and soluble signaling. In this regard, longitudinal studies of pre-cirrhotic MASLD that combine immune profiling of blood and available tissue samples with separate assessments of steatosis, insulin resistance, and fibrogenesis are of potential interest.

### 4.5. Sex and Reproductive Status as Modifiers of Interorgan Regulation

Sex differences in hepatic lipid metabolism may contribute to the modulation of canonical and non-canonical regulatory pathways. The first mechanism is associated with the nature of growth hormone secretion. Pulsatile secretion in male mice induces cyclic activation of STAT5 and the periodic opening of several thousand regions of accessible chromatin in the liver, and a single administration of a physiological dose of the hormone to hypophysectomized animals restored STAT5 binding and chromatin accessibility in 83% of the dynamic male-biased chromatin regions within 30 min [243]. Delivery of constitutively active STAT5b to the livers of male mice reproduces the “female” transcriptional profile [244]. A second mechanism is associated with the hepatic estrogen receptor α (ERα). In mice, ovariectomy altered the expression of 1426 genes when the receptor was intact and only 245 genes when it was silenced in the liver, while in women with MASLD, a shift in the liver transcriptome toward a “male” profile was identified in all 15 subjects aged 51 and older [245]. The regulatory role of sex is further confirmed by its interaction with the genetic background: the effect of the *PNPLA3* p.I148M variant on steatosis and fibrosis is multiplicatively amplified in women (*p* < 10^−10^), and hepatic *PNPLA3* expression in obese women is higher and regulated by an enhancer that binds to ERα [246]. The clinical implications of these differences are stage-specific. According to a meta-analysis of 54 studies, women have a 19% lower risk of MASLD (relative risk (RR) 0.81; 95% CI 0.68–0.97) while having an equal risk of steatohepatitis; however, their risk of advanced fibrosis is 37% higher (1.37; 1.12–1.68), and in samples with a mean age of 50 years or older, it increases to 1.56 (1.36–1.80) [247]. Menopause is associated with an increased likelihood of MASLD (pooled odds ratio 2.37; 95% CI 1.99–2.82; after adjusting for age and metabolic factors, 2.19; 1.73–2.78) [248], and early menopause (before age 50) in matched cohorts, each comprising 20,979 women, increased the five-year risk of the disease (HR 1.322; 95% CI 1.170–1.492) [249].

Non-canonical regulators are directly involved in these differences. In 923 patients with severe obesity and histologically confirmed MASLD, concentrations of FGF21 and leptin were higher, while FGF19 concentrations were lower than control values. Moreover, sex differences in leptin and adiponectin were detected only in the disease group, and the uncoupling of the “FGF21–adiponectin” axis increased as liver damage became more severe [88]. The response to FGF21 itself is sex-dependent: pharmacological administration of FGF21 preserves lean mass in males by enhancing lipid catabolism, whereas it preserves fat mass in females [250]. In a separate study, liver-specific *Fgf21* knockout eliminated ovariectomy-induced hypercorticosteronemia and central obesity but did not restore insulin sensitivity or reduce steatosis [251]. Data stratified by sex are still insufficient: no such analysis has been published for resmetirom, and a post hoc analysis of the SYNERGY-NASH study showed comparable efficacy of tirzepatide in men and women, indicating reproducibility rather than a discrepancy in effect [252].

Pregnancy represents a physiological model of increased lipid load on the liver. Normally, during gestation, total cholesterol and low-density lipoprotein (LDL) cholesterol increase by approximately 30–50%, HDL cholesterol by 20–40%, and triglycerides by 50–100% [253]. Nutrient metabolism in the liver during pregnancy is coordinated by a set of regulators that substantially overlap with the non-canonical signaling molecules discussed above—FGF21, serotonin, leptin, and GLP-1, along with sex steroids and growth hormone [20]. The remodeling of the mother’s hepatic immune response constitutes a separate level of regulation [254]. The dynamics of FGF21 are stage-specific: its concentration is reduced in the first and second trimesters, peaks in the third (*p* < 0.01), and is elevated in preeclampsia (*p* < 0.01) [255]. The clinical significance has been confirmed by a national Swedish cohort. In biopsy-confirmed MASLD, the rate of preterm birth was 16.7% versus 4.7% (adjusted odds ratio 3.41; 95% CI 1.98–5.88); moreover, when compared with overweight and obese women without MASLD, the odds ratio increased to 4.60 (2.00–10.60) without an increase in the incidence of stillbirths or congenital anomalies [256]. According to a meta-analysis of 22 studies (13,641 women), the condition is associated with preeclampsia (odds ratio 2.43; 1.46–4.04), gestational diabetes (3.23; 1.97–5.31), and preterm birth (2.02; 1.44–2.85) [257].

It is significant that, in intrahepatic cholestasis of pregnancy, colonizing mice with the patients’ microbiota alone induced cholestasis, while *Bacteroides fragilis* suppressed FXR signaling through the activity of bile salt hydrolase [258]. That is, the same “gut-liver” axis retains its regulatory role even under these conditions.

Thus, the data presented suggest that sex and reproductive status should be considered modifiers of interorgan axes rather than as confounding factors. At the same time, the predominance of studies in male animals remains a significant limitation on the generalizability of mechanistic conclusions.

## 5. Imbalance Between Canonical and Non-Canonical Regulators in the Pathogenesis of MASLD

### 5.1. Quantitative Imbalance in Lipid Flows

Steatosis arises from a quantitative imbalance between the influx, synthesis, oxidation, and export of hepatic lipids. In a classic study involving the administration of stable isotopes over several days, 59.0 ± 9.9% of the fatty acids in hepatic triglycerides originated from serum non-esterified fatty acids, 26.1 ± 6.7% from de novo synthesis, and 14.9 ± 7.0% from dietary lipids [259]. This ratio is not constant. The proportion of de novo lipogenesis in the hepatic palmitate pool was 11% in individuals with normal body weight, 19% in those with obesity without steatosis, and 38% in those with obesity and MASLD [260], reached 40.7% (interquartile range 32.1–47.5) in fibrotic steatohepatitis [261], and decreased by 67% following a 10% reduction in body weight, whereas the absolute contribution of plasma fatty acids and dietary lipids remained unchanged [262]. It is the intensity of de novo synthesis, rather than the influx of fatty acids, that distinguishes MASLD from obesity without steatosis. However, this does not mean that fatty acid influx ceases to matter. The contribution of adipose tissue to steatosis lies not in an increase in basal lipolysis but in the loss of its suppression by insulin [263,264,265]. The adipose tissue insulin resistance index correlates with fibrosis and hepatic insulin resistance, but these observations are cross-sectional and do not prove causality [266,267].

The compensatory capacity for lipid export from the liver is limited. The secretion of triglycerides in VLDL is approximately doubled in steatosis (24.3 ± 3.1 vs. 11.4 ± 1.1 μmol/min), but this rise plateaus once intrahepatic lipid content exceeds approximately 10%: export continues to increase but no longer keeps pace with influx [268]. In patients with severe obesity, hepatic lipid content inversely correlated with the rate of secretion (r = −0.92) [269], and insulin lost its ability to suppress secretion (by 31.9 ± 17.2% versus 64.7 ± 19.9% in the control group) while glucose production remained suppressed [270]. The increase in lipogenesis in this context is largely determined by substrate load: seven weeks of consuming 80 g of sugar per day doubled the fractional secretion rate of newly synthesized fatty acids (20.8%/day for sucrose and 19.7%/day for fructose versus 9.1%/day in the control group) [271], while a nine-day restriction of fructose in obese children reduced the area under the fractional lipogenesis curve from 68% to 26% [272]. The overall picture shifts the emphasis away from the concept of “selective” hepatic insulin resistance, which preserves the lipogenic branch of the cascade, to a model of substrate-dependent ChREBP activation, which is consistent with direct measurements in patients [41].

### 5.2. Shift in Equilibrium During the Simple Steatosis Stage

It is helpful to view the progression of MASLD as the result of a shift in the balance between canonical (primarily insulin-dependent) and non-canonical signaling pathways. At the stage of simple steatosis, two processes play a leading role: an excessive influx of non-esterified fatty acids (NEFAs) from insulin-resistant adipose tissue, where insulin-mediated suppression of hormone-sensitive lipase is impaired [199,273], and significantly enhanced de novo lipogenesis, whose contribution to the hepatic triglyceride pool increases as the disease progresses (Section 5.1) [273,274]. Increased lipogenesis is maintained by the transcription factors SREBP-1c (insulin-activated) and ChREBP (glucose-activated) under conditions of insulin resistance [37,69,273]. The traditional explanation of this phenomenon—involving “selective” hepatic insulin resistance, in which the insulin cascade loses its ability to suppress gluconeogenesis but retains its ability to stimulate lipogenesis—is not supported by direct measurements in patients; therefore, an interpretation based on substrate regulation is preferred. At the same time, protective non-canonical signals decline. Adiponectin levels decline [47,275], resistance to FGF21 [47,103,276] and leptin [275,277,278] develops, and postprandial secretion of FGF19, which mediates the regulation of lipid metabolism, is attenuated [118,279,280].

### 5.3. Progression to Steatohepatitis and Fibrosis

The progression to steatohepatitis is associated with the activation of proinflammatory and profibrogenic non-canonical mediators, one of which is intestinal serotonin. Intestinal serotonin, acting through the hepatic HTR2A receptor, stimulates lipogenesis [173,174] and, as has been established in models of alcoholic liver disease, is also capable of increasing ER stress [175]. Lipocalin-2 directly activates HSCs [151]. Hyperleptinemia acts similarly [278,281], together, these mechanisms contribute to the progression of fibrosis in steatohepatitis. Concurrent with the activation of profibrogenic stimuli, key antifibrotic mechanisms are lost. For example, the loss of the antifibrotic action of FGF21, which normally limits the activation of HSCs and collagen accumulation [99], and adiponectin, which suppresses the proliferation and migration of HSCs [282,283], releases fibrogenesis from crucial inhibitory control. Portal endotoxemia via the “gut–liver” axis, arising from increased intestinal permeability, is a key factor sustaining inflammation. The action of endotoxin is mediated through the activation of TLR4 on Kupffer cells and hepatocytes, followed by the production of proinflammatory cytokines [206]. Thus, fibrosis is not merely a consequence of lipid accumulation but rather the result of a systemic imbalance in the regulatory network, in which non-canonical axes play a decisive role. This concept explains why the severity of fibrosis may not correlate with the degree of steatosis and justifies the search for therapeutic targets beyond the insulin cascade.

FGF21 levels are elevated in MASLD and continue to rise as the condition progresses to steatohepatitis (386.6 ± 328.9 vs. 297.9 ± 231.5 pg/mL; *p* = 0.009), accompanied by an increase in its hepatic expression [284]. Leptin concentrations rise monotonically across disease stages—the standardized mean difference is 0.64 (95% CI 0.42–0.86) compared with controls and 0.21 (0.02–0.40) when comparing steatohepatitis with simple steatosis [285]. In contrast, adiponectin and FGF19 levels decrease [88,286,287]. Thus, the impairment of protective regulation involves two distinct mechanisms: a true ligand deficiency (adiponectin, FGF19) and tissue resistance in the presence of ligand excess (FGF21, leptin). Distinguishing between them is clinically important, since the first mechanism points to ligand replacement, whereas the second points to restoration of signal transduction.

The localization of tissue resistance to FGF21 and leptin has not been fully established, and the available data do not confirm that it is caused by changes in the hepatic receptors for these hormones—FGFR1c/β-Klotho and LepR, respectively. In obesity, the expression of β-Klotho, FGFR1, and FGFR3 in the liver is elevated, whereas in visceral and subcutaneous adipose tissue, β-Klotho levels are reduced [288]. Quantitative assessment of β-Klotho protein in 28 biopsies revealed an association of its reduction with lobular inflammation only, but not with steatosis or fibrosis; moreover, expression persisted even in later stages [289]. Data on hepatic adiponectin receptors are conflicting: in one study, AdipoR2 expression in steatohepatitis was lower than in simple steatosis [290], while in another, expression of both receptors increased with histological progression [291]. Adipose tissue remains the most likely site of dysfunction, where both the suppression of β-Klotho by tumor necrosis factor α [292] and an increase in SOCS3, which correlates with insulin resistance [293,294], have been demonstrated. A reproducible intrahepatic mechanism involves disruption of the JAK2/STAT3 pathway during endoplasmic reticulum stress, a process common to both leptin and interleukin-6 [295]. However, direct evidence of simultaneous desensitization of multiple hormonal pathways in human hepatocytes has not been obtained. Causality has also not been established and partly points in the opposite direction: according to Mendelian randomization data, changes in FGF21 are a consequence of the disease, not its cause [296]. Genetically determined higher leptin levels are associated with a reduced risk of MASLD [297], and no causal effect of adiponectin on insulin sensitivity has been identified [298]. Associations between the adipokine profile and disease stage should therefore be regarded as marker associations rather than as evidence of a pathogenic relationship.

### 5.4. Spatial and Cellular Organization of the Imbalance

The imbalance described occurs unevenly throughout the organ; therefore, its accurate description requires spatial coordinates. The hepatic lobule is functionally compartmentalized. According to Halpern et al., about half of liver genes exhibit a zonal expression profile, with many genes showing peak expression in the midlobular region [299]. In the human liver, 1141 out of 1724 hepatocyte-specific genes were found to be zonally expressed (q < 0.25), with some genes showing peak expression in the midzonal region, including *HSD17B13*, *C6*, *KLKB1*, *LIPC*, *HGD*, and *SDC1* [300]. In humans, this pattern differs from that in mice—key metabolic functions are shifted toward the pericentral region, such as *PCK2*, *SLC2A2*, *HNF4A*, *FASN*, *APOA5*, *GPAM*, *ACSL5*, *DPP4*, as well as *NAGS*, *CPS1*, *OTC*, and *ASL* [300]. In hepatocytes at an early stage of steatosis, a decrease in nuclear-encoded mitochondrial proteins (*NDUFA9*, *NDUFS4*, *NDUFS8*) has been observed, accompanied by a compensatory increase in mitochondrial genome transcripts (*MT-ND1*, *MT-ND4L*) [300]. Therefore, the zonal coordinates obtained in mice cannot be directly extrapolated to humans. The localization of steatosis also depends on the genetic background: in homozygous carriers of the *PNPLA3* p.I148M variant, periportal hepatocytes exhibited higher lipid metabolism and lower mitochondrial activity, and in a validation group of 100 biopsies, the predominance of periportal steatosis, inflammation, and fibrosis was confirmed [301]. The notion of a consistently pericentral (third zone) distribution of steatosis thus requires qualification; in an experimental MASH model, the opposite redistribution of triglycerides, diacylglycerols, and sphingolipids from the pericentral to the periportal region has been demonstrated [302].

It is also important to note the change in the cellular state itself in MASLD. According to sequencing data from the nuclei of 47 human biopsies, as MASLD progresses, hepatocytes lose their zonal specialization, the biliary tree is reorganized, and some hepatocytes directly transdifferentiate into cholangiocytes (with the reverse direction—cholangiocyte → hepatocyte—predominating), and this transition depends on the PI3K–AKT–mTOR cascade [303]. The acquired plasticity of the epithelium is thus linked to the same insulin signaling axis that regulates lipogenesis [303]. Longitudinal multi-omics data confirm that chronic metabolic stress activates developmental and tumor-like programs in non-transformed hepatocytes while simultaneously weakening their mature functional identity, and the cells organize into stable multicellular communities [304]. The imbalance in regulators thus manifests not only as altered metabolic fluxes but also as changes in cellular identity, which explains why the response to the same signal depends on the cell’s initial state [303,304].

It is also important to note the role of local immune and mesenchymal niches. In the human liver, fibrosis is accompanied by an expansion of the population of TREM2^+^CD9^+^ scar-associated macrophages, which originate from blood monocytes and possess a profibrogenic phenotype [305], while the endothelium of the fibrotic niche acquires ACKR1^+^ and PLVAP^+^ phenotypes, topographically restricted to the scar area [305]. Lipid-associated macrophages form in response to local lipid overload and are located predominantly in steatotic areas and near the bile ducts [306]. The 2025 atlas confirms the accumulation of TREM2^+^S100A9^+^ macrophages, monocytic myeloid-derived suppressor cells (M-MDSCs), and hepatic regulatory T cells (Tregs) as MASH progresses, accompanied by a decrease in the cytotoxic function of CD8^+^ T cells and their acquisition of an exhausted phenotype at the fibrosis stage [307]. At the same time, the role of these cells is stage-specific and is not limited to a profibrogenic effect: *TREM2* deficiency in hematopoietic cells exacerbated steatohepatitis and fibrosis [308], TREM2 on lipid-associated macrophages (LAMs) or LAM-like Kupffer cells (KCs) is essential for the removal of dying cells and tissue repair [309], and the hepatocyte growth factor (HGF) secreted by LAMs exerts a hepatoprotective effect via the HGF–MET axis [310]. The mesenchymal component is also heterogeneous: in humans, GPC3^+^ and DBH^+^ stellate cell subpopulations have been identified [311], and in MASH, the primary source of the matrix is activated subclusters with a reproducible core of activation genes (*GAS7*, *SPON1*, *SERPINE1*, *LTBP2*, *KLF9*, *EFEMP1*) [312], and the profibrogenic role of PAI-1 (encoded by *SERPINE1*) has been confirmed in three-dimensional models of the human liver [312]. The topographic hierarchy of portal and central stellate cells has been described in mouse models but has not been replicated in humans with MASH, which limits the generalizability of this model. This picture is complemented by early capillarization of the sinusoidal endothelium, which contributes to the development of steatosis and the loss of resting state in stellate cells [313]. The practical implication is that bulk measurements averaged across the organ—such as fat content, regulator expression, and biomarker concentration—average out the states of different niches and thus provide only a limited reflection of the local events that drive disease progression.

### 5.5. Integrative Model

The data presented allow the progression of MASLD to be described as a shift in the dominant mechanisms rather than as a linear intensification of a single process (Table 2). At the stage of simple steatosis, a quantitative imbalance in fluxes predominates: the influx of substrate from insulin-resistant adipose tissue and substrate-dependent lipogenesis exceed the capacity for oxidation and export. In steatohepatitis, qualitative characteristics come to the fore—the composition of lipid pools, lipotoxic damage, and the formation of localized immune niches—while the total fat content ceases to linearly reflect disease activity. At the fibrosis stage, the balance between the synthesis and degradation of the extracellular matrix becomes decisive, which metabolic interventions influence only indirectly.

## 6. Tissue-Specific Signal Integration: Distinguishing Between Metabolic and Fibrogenic Effects

The classification of regulators into canonical and non-canonical categories describes the origin of the signal but does not predict its outcome in the liver. The same mediator can simultaneously reduce steatosis and enhance fibrogenesis, while a reduction in liver fat content may not be accompanied by regression of fibrosis. The outcome is determined by three parameters: the cells or tissues receiving the signal (hepatocyte, stellate cell, adipocyte, neuron, immune cell), the regulated process (lipogenesis, β-oxidation, lipoprotein export, matrix synthesis or degradation), and the endpoint by which the effect is assessed (triglyceride content, lipotoxic damage, histological activity, stage of fibrosis).

A key implication of this concept is that triglyceride accumulation and lipotoxic damage to hepatocytes are related but not identical events [317]. A clear illustration of this is the regulation of interactions between lipid droplets and mitochondria via the phosphorylation of PLIN5 at residue S155. The nonphosphorylatable PLIN5-S155A variant enhances these interactions, increases triglyceride accumulation in lipid droplets, and simultaneously reduces lipid peroxidation, whereas the phosphomimetic PLIN5-S155E reduces the number and size of droplets but impairs the redox balance. Thus, the increase in triglyceride content may reflect adaptive fatty acid storage rather than lipotoxic damage.

The second implication concerns fibrosis. A reduction in steatosis is not equivalent to an antifibrogenic effect: fibrosis regression requires not only the suppression of collagen synthesis but also the activation of its degradation [316]. Inhibition of acid ceramidase increases ceramide levels, activates the PKCα–ERK1/2–AP1 signaling cascade in hepatic stellate cells, and induces MMP-1, thereby enhancing collagen degradation. In CCl_4−_ and FPC-induced MASH models, this reduced fibrosis without significantly altering steatosis, lobular inflammation, or hepatocyte ballooning [316]. Thus, signal integration involves three levels—substrate (the ratio of receptor signal to substrate availability), interorgan (the distribution of receptor competence among organs), and intrahepatic (the distribution of lipids among cells and organelles).

The first level of integration determines whether the lipogenic program is implemented via a receptor-mediated (transcriptional) or a substrate-mediated mechanism. In patients with obesity and NAFLD, a proximal block in insulin signaling has been identified. Phosphorylation of the insulin receptor kinase (IRK) and AKT in response to glucose did not increase, whereas in control subjects without steatosis, it did increase [41]. Glucose-stimulated/insulin-mediated de novo lipogenesis in patients with NAFLD was not increased but decreased, whereas fructose-stimulated lipogenesis at low insulin levels remained intact [41]. The expression of the lipogenic transcription factor ChREBPβ was constitutively elevated in patients with NAFLD, indicating substrate-dependent rather than insulin-dependent activation of the lipogenic program [41]. These data limit the applicability of the model of selective hepatic insulin resistance [38,41].

The substrate supply for lipogenesis changes even without the involvement of the transcriptional program. In isotope-labeling experiments in mice, microbial acetate produced during fructose excess was incorporated into fatty acids via hepatic ACSS2, independent of ACLY [318]. ACSS2 deficiency or depletion of the microbiota by antibiotics significantly reduced the incorporation of fructose carbon into hepatic acetyl-CoA and palmitate, while the expression of lipogenic genes (*Mlxipl*, *Fasn*, *Acaca*) remained intact [318]. Thus, lipogenesis is regulated at two relatively independent levels—the transcriptional level (via ChREBP) and the substrate level (via acetyl-CoA availability)—and intervention at one level does not replicate the effect of the other.

The second level determines which organ must receive the signal in order to elicit the desired effect in the liver. The most striking example comes from tissue-specific interventions in MASH mouse models, which dissociated the effects of FGF21. To reduce hepatic triglycerides and fibrosis, the β-Klotho co-receptor (a protein encoded by the *KLB* gene) was required in glutamatergic neurons of the central nervous system, whereas its expression in hepatocytes was necessary and sufficient only for reducing hepatic cholesterol. Knocking out β-Klotho in adipocytes did not abolish the protective effect, although it prevented the increase in adiponectin [98]. A single hormone thus exerts different effects through different tissues, and an increase in adiponectin is not a mandatory mediator of its action. Incretins provide a counterexample: in human hepatocytes and hepatic stellate cells (HSCs), liraglutide, acylated glucose-dependent insulinotropic polypeptide (Acyl-GIP), and the dual GLP-1/GIP agonist (MAR709) did not reduce lipid accumulation or TGF-β (transforming growth factor-β)-induced expression of fibrogenic markers, including *COL1A1*, *COL1A2*, *ACTA2*, and *TIMP-1* [319]. The clinical effect of this class appears to be mediated by systemic changes in body weight and metabolism, rather than by a direct action on liver cells. This level of integration explains why a drug’s efficacy cannot be predicted based on its target: receptor competence is distributed unevenly among organs, and it is precisely this distribution that limits the outcome.

The third level determines in which cell and in which organelle a change in a lipid metabolite occurs. Here, tissue specificity is most clearly expressed. In hepatocytes, targeted inhibition of serine palmitoyltransferase (SPT) via knockdown of the *Sptlc2* gene (which encodes the SPTLC2 subunit) using lipid nanoparticles with small interfering RNA (LNP-siRNA) reduced steatosis, inflammation, and fibrosis in MASH models [320]. In hepatic stellate cells, a different intervention that increased rather than decreased ceramide levels was beneficial. Inhibition of acid ceramidase (aCDase) increased ceramide levels, activated the PKCα–ERK1/2–AP1–MMP-1 signaling cascade, and accelerated collagen degradation and fibrosis regression [316]. It is important to note that this level of integration can operate not only at the cellular but also at the organellar scale. Increased interactions between lipid droplets and mitochondria via PLIN5 were accompanied by triglyceride accumulation and a reduction in oxidative stress. The PLIN5-S155A variant increased the number of contacts between mitochondria and lipid droplets, raised triglyceride levels, and reduced lipid peroxidation [317]. The same fat content can reflect either adaptive fat storage or a lipotoxic state [317]. Thus, it is not only the direction of the metabolite change that matters, but also the cell in which this change occurs.

Consequently, lipid fluxes, hepatocyte damage, and extracellular matrix turnover must be evaluated separately. A reduction in fat content does not prove fibrosis regression, and a decrease in collagen does not indicate a primary cellular target. The effect of a single signal depends on three parameters: substrate availability, receptor localization, and the target cell. This means that a reduction in fat content and a reduction in fibrosis may have different molecular mechanisms—and, accordingly, require different interpretations when evaluating therapy.

## 7. Diagnostic and Therapeutic Perspectives

The elucidation of non-canonical pathways has already translated into clinical solutions. Steatosis indices, particularly FLI, are being actively studied as noninvasive predictors of cardiovascular outcomes in the context of CKM syndrome [32,321]. Circulating FGF21 has established itself as a biomarker of metabolic syndrome [322,323,324,325] and of individual components of CKM syndrome, such as obesity [326,327], as well as a potential biomarker of insulin resistance and type 2 diabetes [94], and vascular disease [322,328,329,330,331]. Evidence is also gradually accumulating regarding the renal component of CKM syndrome. For example, genetically elevated FGF21 activity, associated with higher glomerular filtration rates, sodium excretion, and reduced albuminuria [332], is supported by meta-analytic data showing that elevated circulating FGF21 predicts the development of chronic kidney disease and adverse renal outcomes in patients with type 2 diabetes [333]. At the same time, the role of FGF21 specifically in risk stratification for CKM syndrome has not yet been established. There are no large prospective studies in which FGF21 has been evaluated as an independent or component biomarker of CKM stages and transitions between stages.

The role of adiponectin as a noninvasive biomarker for risk stratification within the context of CKM remains poorly understood and requires targeted prospective studies. Existing data indicate its association with specific components of CKM—diastolic dysfunction [334,335]—and the progression of chronic kidney disease [336,337,338,339,340]. Although the identification and validation of non-canonical biomarkers are ongoing, the greatest breakthrough to date has been achieved specifically in the field of targeted pharmacotherapy, such as resmetirom for the treatment of MASH with fibrosis, as well as successful trials of GLP-1 receptor agonists, FGF21 analogs, and other drugs [341,342,343].

### 7.1. Thyroid Hormone Receptor β Agonists

Resmetirom became the first drug to receive accelerated approval from the U.S. Food and Drug Administration (FDA) in March 2024 for adult patients with noncirrhotic MASH and moderate or advanced fibrosis (stages F2–F3) in combination with diet and physical activity [166,344,345]. By selectively activating the hepatic thyroid hormone receptor β (THR-β), resmetirom enhances mitochondrial β-oxidation of fatty acids and inhibits de novo lipogenesis, leading to a significant reduction in intrahepatic lipid content [344,346]. In the MAESTRO-NASH study, treatment with resmetirom at doses of 80 and 100 mg resulted in statistically significant clinical improvement and an increase in the rate of NASH resolution with no worsening of fibrosis in 25.9% and 29.9% of patients receiving 80 and 100 mg, respectively, versus 9.7% in the placebo group, and fibrosis improvement by at least one stage with no worsening of the NAFLD activity score in 24.2% and 25.9% versus 14.2% [345]. In addition to histological and radiological benefits, resmetirom therapy is associated with a significant improvement in quality of life. Patients receiving the drug showed a reduction in the severity of abdominal symptoms, as well as a decrease in disease-related anxiety and health-related distress, according to questionnaire data [347,348]. These beneficial effects are observed across the entire spectrum of MASLD—from early MASH to compensated cirrhosis—and are most pronounced in patients who have achieved a 30% or greater reduction in the liver fat fraction [348].

### 7.2. PPAR Agonists

The canonical regulators of fatty acid oxidation, PPARα and PPARδ (Section 2), are pharmacological targets, and their action profiles are determined by their isotype specificity: PPARα regulates lipid catabolism, PPARβ/δ exerts an anti-inflammatory effect and enhances glucose uptake by muscles, and PPARγ increases insulin sensitivity and has a direct antifibrogenic effect in stellate cells [349]. Selective PPARα agonists (fibrates) did not reduce steatosis; the PPARγ agonist rosiglitazone improved steatosis but did not resolve steatohepatitis, whereas the PPARδ agonist seladelpar did not reduce liver fat content [349]. The modest histological results of selective agonists served as the rationale for the simultaneous activation of all isotypes [349]. The pan-agonist lanifibranor, in the Phase II NATIVE trial (*n* = 247, 24 weeks), achieved the primary endpoint—a reduction of at least 2 points in the activity component of the steatosis, activity, and fibrosis (SAF) score without worsening of fibrosis—in 55% of patients at a dose of 1200 mg versus 33% in the placebo group (*p* = 0.007); at a dose of 800 mg—in 48% versus 33% (*p* = 0.07) [350]. Resolution of steatohepatitis without worsening of fibrosis was observed in 49% and 39% versus 22%, and improvement in fibrosis by at least one stage was observed in 48% and 34% versus 29% [350]. Metabolic parameters improved regardless of the presence of type 2 diabetes (T2D); however, treatment was accompanied by an average weight gain of 2.5 kg [351]. In patients with T2D and MASLD, lanifibranor at a dose of 800 mg reduced intrahepatic triglyceride (IHTG) levels by 44% compared with 12% in the placebo group and increased adiponectin concentration 2.4-fold, linking this class to the adipokine axis [352]. In the Phase III NATiV3 trial (NCT04849728), the 72-week follow-up period has not yet been completed, and the results have not been published [353].

Selective and dual agonists yielded more modest results. The selective PPARα modulator pemafibrate failed to meet the primary endpoint—a change in liver fat content as measured by magnetic resonance imaging–proton density fat fraction (MRI-PDFF)—in the 72-week PEMA-FL study (*n* = 118) (−5.3% vs. −4.2%; *p* = 0.85); however, compared with placebo, it reduced liver stiffness as measured by magnetic resonance elastography (MRE) by 5.7% at week 48 and by 6.2% at week 72 [354]. In the PROMINENT study (*n* = 10,497) in patients with type 2 diabetes, hypertriglyceridemia, and low HDL cholesterol, the drug reduced triglyceride levels by 26.2% and remnant cholesterol by 25.6%, but had no effect on the incidence of cardiovascular events (HR 1.03; 95% CI 0.91–1.15) and was terminated early for futility [355]. The PPARα/γ dual agonist saroglitazar, in a randomized, double-blind Phase II trial (*n* = 106, 16 weeks), reduced ALT levels by 45.8% at a dose of 4 mg, compared with a 3.4% increase in the placebo group (*p* < 0.001), and decreased liver fat content by 19.7% [356]. The drug is approved in India for this indication, and in a Phase IV observational program involving 500 patients, liver stiffness decreased from 10.1 to 8.0 kPa after 24 weeks [357]. The PPARα/δ agonist elafibranor did not demonstrate efficacy in steatohepatitis: in the prematurely terminated Phase III RESOLVE-IT trial (NCT02704403), resolution of MASH without worsening fibrosis was achieved in 19.2% versus 14.7%. The drug was subsequently repositioned for primary biliary cholangitis, where the biochemical response rate was 51% versus 4% [358,359,360,361]. Thus, within a single class, the therapeutic niche is determined not by the mechanism per se, but by the tissue and the disease in which the relevant pathway is a rate-limiting determinant of disease progression.

The longest-standing clinical experience is with the peroxisome proliferator-activated receptor γ (PPARγ) agonist pioglitazone. In the PIVENS study (96 weeks), resolution of steatohepatitis was observed in 47% of patients versus 21% (*p* = 0.001), although the predefined significance threshold for the primary histological endpoint was not met (34% versus 19%; *p* = 0.04) [362]. A meta-analysis of eight biopsy-controlled studies (516 patients) confirmed an improvement in advanced fibrosis (odds ratio 3.15; 95% CI 1.25–7.93) and resolution of steatohepatitis (3.22; 2.17–4.79); all effects were attributable to pioglitazone rather than rosiglitazone. In individuals without diabetes, the benefits persisted: the odds ratio for improvement in advanced fibrosis was 2.95 (95% CI 1.04–10.90), and for resolution of steatohepatitis, 3.40 (1.95–5.93) [363].

The side effects of PPAR agonists are due to their mechanism of action. Activation of PPARγ enhances amiloride-sensitive sodium reabsorption in the collecting ducts, causing fluid retention and edema [364]. It is also associated with weight gain [363], an increased risk of fractures in women [365], and a moderate increase in the risk of bladder cancer with long-term use [366]. PPARα agonists are characterized by a reversible increase in creatinine. In particular, the dual PPARα/δ agonist elafibranor has been shown to cause a mild, reversible increase in serum creatinine [367].

A network meta-analysis of 29 studies with biopsy-assessed outcomes (9324 patients) places PPAR agonists in the middle of the range: for MASH resolution, the RR was 2.29 (95% Bayesian credible interval 1.41–4.03) for pioglitazone and 1.93 (1.30–3.02) for lanifibranor; however, this network meta-analysis found no significant effect for either pioglitazone or lanifibranor in terms of improving fibrosis by at least one stage—unlike resmetirom, semaglutide, and tirzepatide [368]. This discrepancy with an earlier meta-analysis, in which pioglitazone improved advanced fibrosis (odds ratio 3.15; 95% CI 1.25–7.93) and fibrosis of any stage (1.66; 1.12–2.47) [363], underscores the dependence of the findings on the chosen endpoint, fibrosis stage, and evidence synthesis method. Thus, PPAR agonists remain a rational choice when insulin resistance and the metabolic component predominate, but their antifibrogenic effect is not universal and requires further clarification in prospective studies, and their use is limited by their safety profile.

### 7.3. Analogs of Fibroblast Growth Factor 21 (FGF21)

Several FGF21 analogs, including efruxifermin [369,370,371], pegbelfermin [371,372,373], and efimosfermin alfa [371,374,375,376,377], are currently undergoing Phase II–III clinical trials and are showing promising results in reducing steatosis, inflammation, and fibrosis in MASH [371,378,379].

Efruxifermin is an FGF21 analog consisting of a bivalent Fc–FGF21 fusion protein. In the randomized, placebo-controlled BALANCED trial in patients with NASH (F1–F3), 16 weeks of treatment with efruxifermin resulted in a significant reduction in hepatic fat fraction, markers of liver damage, and fibrosis [370]. A subsequent network meta-analysis confirmed that efruxifermin (50 mg) nearly doubles the likelihood of fibrosis improvement by at least one stage without worsening MASH (RR 2.23; 95% CI 1.32–3.74) [380]. Beneficial effects were also observed in patients with compensated cirrhosis [369], which, combined with data on improvements in metabolic parameters [371], positions efruxifermin as a promising candidate for the treatment of the full spectrum of MASLD.

Pegbelfermin, a pegylated analog of fibroblast growth factor 21 (FGF21), has completed Phase 2b clinical trials (FALCON 1, FALCON 2) in patients with MASH and fibrosis, including compensated cirrhosis. Although the primary histopathological endpoints in these studies were not met [372,373], in exploratory analyses, the drug demonstrated promising results regarding key components of the pathogenesis of MASLD. Specifically, a reduction in hepatic steatosis was observed based on noninvasive imaging data, along with a decrease in markers of fibrosis and fibrogenesis (MRE and N-terminal propeptide of type III collagen (PRO-C3)), as well as improvements in indicators of hepatocellular damage and inflammation (ALT, aspartate aminotransferase (AST)) [372,373]. A meta-analysis confirmed pegbelfermin’s ability to significantly increase adiponectin levels and reduce PRO-C3 concentrations. However, it did not reveal a statistically significant effect on liver stiffness as measured by MRE or on transaminase levels compared with placebo [381]. Nevertheless, according to a network meta-analysis, pegbelfermin is inferior to other FGF21 analogs (such as efruxifermin and pegozafermin) in its ability to induce fibrosis regression based on biopsy data [380], which defines its potential niche as a drug that acts more on metabolic and inflammatory signatures than on established fibrosis.

Efimosfermin alfa, an analog of FGF21 with a long half-life, is currently undergoing Phase 3 clinical trials (ZENITH-2; NCT07221188) [376]. A Phase 2a study demonstrated that, over 12 weeks of treatment, the drug produced a marked reduction in hepatic steatosis: 89% of participants achieved a 30% or greater reduction in the fat fraction as measured by MRI-PDFF [377]. In a 24-week, biopsy-controlled Phase 2b study in patients with MASH and F2–F3 fibrosis, efimosfermin alfa produced a statistically significant improvement in histological endpoints [374,375]. Thus, the drug demonstrates efficacy against all three key components of MASLD—steatosis, inflammation, and fibrosis. However, these data were obtained in a limited sample and require confirmation in the ongoing Phase 3 trial.

Aldafermin (NGM282), an FGF19 analog, has been shown in placebo-controlled Phase 2b trials to significantly reduce liver fat content, transaminases, and markers of fibrogenesis, such as PRO-C3 and ELF, in patients with noncirrhotic MASH [382,383]. An important component of its mechanism of action is the potent suppression of the synthesis of hydrophobic, highly cytotoxic bile acids, as demonstrated in detail in a pooled analysis of Phase 2 studies in patients with NASH and primary sclerosing cholangitis [384]. Aldafermin’s therapeutic potential extends to the cirrhosis stage as well. In the ALPINE-4 study of patients with compensated NASH-cirrhosis, 48-week therapy led to a statistically significant improvement in the primary endpoint—the enhanced liver fibrosis (ELF) score—as well as favorable trends in PRO-C3 and transaminases [385]. Despite these encouraging results, aldafermin remains an experimental therapy and is not an established standard of care.

### 7.4. Restoration of Adipokine Signaling

Hypoadiponectinemia and leptin resistance are the two most reproducible adipokine abnormalities in MASLD, and both are, in principle, amenable to pharmacological correction. Adiponectin itself, however, is unsuitable as a drug. First, it circulates in plasma at micromolar concentrations that are exceptionally high for a hormone, accounting for up to 0.05% of total serum protein [386], which poses significant challenges for determining the appropriate dosage and maintaining therapeutic levels upon exogenous administration. Second, in the body it exists as a complex mixture of isoforms: trimers (low-molecular-weight form), hexamers (medium-molecular-weight form), and high-molecular-weight (HMW) multimers consisting of 12–18 subunits [386,387]. It is the HMW form that is considered the most biologically active, particularly in terms of insulin-sensitizing effects [386]. This accounts for the varying biological activity of different oligomers. Finally, a key obstacle is that the production of recombinant human protein in therapeutically significant quantities and with the correct multimeric structure remains an unresolved technological challenge. Unlike bacterial systems, only production in mammalian cells yields a functionally active protein due to complex post-translational modifications (hydroxylation and glycosylation of lysine in the collagen domain). However, this approach is associated with high costs and heterogeneity of the final product [387]. This is precisely why therapeutic modulators based on the adiponectin protein itself are currently unavailable [387]. Consequently, research efforts have shifted toward the development of peptide and small-molecule ligands (agonists) for the AdipoR1 and AdipoR2 receptors, which has become the primary strategy in this field [387]. These compounds, such as the peptide agonist ADP355 [387,388] and the small molecule AdipoRon [388], replicate a key step in the receptor response—the activation of AMPK. Furthermore, given that the AdipoR1/2 receptors themselves possess intrinsic ceramidase activity, hydrolyzing ceramides and thereby improving insulin sensitivity, it is hypothesized that their agonists may also modulate this pathway [389,390].

The importance of the ceramidase pathway has been confirmed genetically. Induced overexpression of AdipoR1 or AdipoR2 in hepatocytes or adipocytes of adult mice increases ceramidase activity, improves systemic glucose metabolism and hepatic insulin sensitivity, and prevents the development of steatosis, whereas in the context of adiponectin knockout, these benefits are completely lost [128]. The receptor, therefore, functions not as an autonomous enzyme but as a ligand-dependent switch for sphingolipid metabolism, which makes its agonism a rational therapeutic target.

AdipoRon is an orally active non-peptide AdipoR1/AdipoR2 agonist, first characterized as a compound that replicates the metabolic effects of adiponectin and increases the lifespan of *db*/*db* mice on a high-fat diet [391]. In models of steatohepatitis, it acts on all three histological components of the disease. In mice fed a Western diet or a methionine-choline-deficient diet, AdipoRon reduced liver damage, steatosis, lobular inflammation, and collagen deposition; proteomic analysis identified the suppression of ER stress as the central mechanism, and pharmacological reactivation of this stress with cinchonine completely reversed the hepatoprotective effect [392]. In carbon tetrachloride-induced liver injury, AdipoRon dose-dependently reduced the levels of hydroxyproline and hyaluronic acid, as well as the expression of TGF-β1, α-SMA, and COL1A1, which is attributed to the deactivation of HSCs [393]. An additional antifibrotic mechanism has been described. Inhibition of ATF4-dependent glutaminolysis and the biosynthesis of serine and glycine deprives activated HSCs of the biosynthetic precursors required for collagen synthesis [394].

The peptide approach is represented by the compound ADP355—a decapeptide that mimics the active domain of globular adiponectin. In mice with established fibrosis induced by carbon tetrachloride, a two-week administration of ADP355 delivered via nanoparticles reduced transaminase activity and hydroxyproline levels, decreased the severity of fibrosis as assessed by histological examination, and suppressed the expression of α-SMA, TGF-β1, connective tissue growth factor (CTGF), and TIMP-1, accompanied by increased phosphorylation of AMPK and endothelial nitric oxide synthase (eNOS) [395]. The antifibrotic activity of AdipoR agonists is not limited to the liver. In models of systemic sclerosis, they suppressed the fibrotic response of fibroblasts and also prevented and reversed experimental skin fibrosis [396], indicating a common mechanism of action. A systematic review of preclinical studies confirmed the reproducibility of the effects of AdipoRon and ADP355 in models of MASLD and steatohepatitis—reduction in steatosis, inflammation, and fibrosis, as well as improvements in insulin sensitivity and mitochondrial function [397]. At the same time, however, it noted a complete lack of clinical data [397]. It is precisely this gap that defines the current status of this field. No adiponectin receptor agonist has entered clinical trials for MASLD, and the pharmacological properties of first-generation compounds remain the subject of ongoing study and development. Analogs of AdipoRon with enhanced anti-inflammatory activity in models of steatohepatitis [398] and with more pronounced AMPK activation have been described [399]. Until data on the pharmacokinetics, safety, and selectivity of AdipoRon and ADP355 in humans are available, it is more appropriate to view them as target validation tools rather than as candidates ready for clinical development.

Leptin resensitization follows the opposite therapeutic logic. In typical obesity and MASLD, the problem lies not in a deficiency but in an excess of leptin, to which a complex form of resistance develops, affecting both central and peripheral mechanisms of the hormone’s action [400,401]. Indeed, central leptin resistance at the hypothalamic level is recognized as a key link in the pathogenesis, disrupting the regulation of appetite and energy balance. However, a growing body of evidence, including direct studies in patients, points to the critical role of peripheral resistance. In particular, it has been shown that activation of the “leptin–leptin receptor–SOCS3” axis in visceral adipose tissue and the liver directly correlates with disease progression from simple steatosis to MASH, exacerbating insulin resistance and inflammation [294]. Replacement therapy offers no prospect here. Signal transmission must be restored. The problem of leptin resistance is complex in nature, and one of the molecular mechanisms contributing to it is the negative regulation of the leptin receptor (LepR) by the enzyme histone deacetylase 6 (HDAC6). It has been shown that HDAC6 physically interacts with LepR, deacetylates it, and thereby reduces receptor activity, while pharmacological inhibition of HDAC6 can disrupt this complex and enhance leptin signaling [402]. Direct evidence for this central mechanism comes from the fact that, in diet-induced obese mice, HDAC6 inhibitors capable of crossing the blood–brain barrier caused a significant reduction in food intake and body weight without loss of muscle mass, whereas non-penetrating analogs, as well as *Hdac6* deletion in Agouti-related protein (AgRP) neurons, did not produce such an effect—which localizes the therapeutically significant target in the central nervous system [402]. Despite these compelling data, the question of whether central (hypothalamic) or peripheral (e.g., in adipose tissue) dysregulation of this mechanism is the key link in leptin resistance remains a subject of scientific debate. While some studies consider central inhibition of HDAC6 to be the primary mechanism for restoring leptin sensitivity [402], others provide evidence that peripheral HDAC6 blockade is what actually triggers central sensitization [144]. An alternative pathway is combinatorial in nature: the combination of an optimized leptin analog with exendin-4 or FGF21 restores the leptin response in mice with diet-induced obesity [403]. These experimental strategies share a common goal—to restore signaling blocked by leptin resistance. Fundamental clinical evidence that restoring leptin signaling itself can exert a potent hepatoprotective effect has been obtained in cases of hypoleptinemia, where the problem lies not in resistance but in an absolute deficiency of the hormone. Thus, among patients with congenital and acquired lipodystrophy, 86% of cases initially met the criteria for steatohepatitis, whereas after an average of 26 months of metreleptin therapy, only 33% did; the mean steatosis score decreased from 1.8 to 0.9, the hepatocyte ballooning score decreased from 1.2 to 0.4, and the NAFLD activity score (NAS) decreased by 44.2% [404]. Long-term follow-up confirmed the sustained metabolic effect [405], and post-marketing surveillance data confirmed an acceptable safety profile [406], although the formation of neutralizing antibodies against the drug remains a clinically significant issue [407].

From a mechanistic standpoint, leptin’s antisteatotic effect in humans mirrors the central pathway described above. A single injection of metreleptin in patients with lipodystrophy increased hepatic secretion of triglycerides in VLDL1 by 75% (a mean increase of 219 ± 149 mg/h relative to placebo) without an immediate change in intracellular lipid content, and in a female patient who received metreleptin after liver transplantation—that is, under conditions of autonomic denervation of the organ—improvements in carbohydrate and lipid metabolism were not accompanied by regression of steatosis [408]. This confirms the neuronal nature of the effect and, at the same time, delineates its limits. In obesity with hyperleptinemia, systemic enhancement of leptin signal is not only ineffective but also potentially harmful, since at the periphery, leptin acts as a profibrogenic factor with respect to HSCs. Thus, therapeutic promise lies with centrally selective resensitizers rather than with increasing the systemic leptin load.

### 7.5. Incretin Therapy

Incretin medications have a multifaceted effect on MASLD, which is mediated both through powerful indirect effects and through direct effects on the liver. The key indirect mechanisms include significant weight loss, restoration of insulin sensitivity, and, as a result, a reduction in the flow of non-esterified fatty acids from adipose tissue to the liver [409,410]. In addition, activation of GLP-1 receptors directly suppresses hepatic de novo lipogenesis, reduces hepatocyte production and secretion of VLDL and intestinal production and secretion of chylomicrons, and exerts independent anti-inflammatory effects, contributing to the resolution of steatohepatitis [410]. The addition of a glucagon component fundamentally alters the mechanism of action. Unlike GLP-1 receptors, glucagon receptors (GCGRs) are abundantly expressed directly on hepatocytes, and their activation directly stimulates mitochondrial β-oxidation of fatty acids, inhibits de novo lipogenesis, and increases energy expenditure [411]. It is precisely these direct hepatotropic effects that explain the significantly more pronounced reduction in hepatic fat fraction observed with dual agonists compared with GLP-1 mono-agonists, even with comparable body weight loss. For example, in a direct comparative study, efinopegdutide (GCGR/GLP-1) demonstrated a significantly greater reduction in liver fat content than semaglutide, which is directly attributed to the direct action of the glucagon component on hepatocytes [411]. A third, less obvious mechanism of action occurs along the “gut–liver” axis. In diabetic mice, tirzepatide altered the composition of the microbiota by increasing the proportion of *Akkermansia* and shifted the bile acid profile toward FXR antagonists while reducing the expression of intestinal FXR [412]. This example illustrates a broader pattern. The interaction between GLP-1 receptor agonists and the gut microbiota is bidirectional and is the subject of active research. A systematic review by Gofron et al., which synthesized 38 preclinical and clinical studies, showed that drugs in this class significantly modulate the composition of the microbiota [413]. In particular, liraglutide and semaglutide promote an increase in the abundance of *Akkermansia muciniphila* and other taxa associated with improved metabolic health [414,415]. In turn, microbial metabolites, such as short-chain fatty acids and secondary bile acids, can stimulate endogenous GLP-1 secretion, thereby closing the feedback loop [414]. These observations suggest that the microbiota can serve both as a biomarker of response to therapy and as a potential target for enhancing it, opening up prospects for personalized approaches to the treatment of MASLD [416].

Semaglutide became the second drug, after resmetirom, to receive regulatory approval for MASH. In an interim analysis of the Phase III ESSENCE study (800 of 1197 randomized patients, 72 weeks), resolution of steatohepatitis without worsening of fibrosis was achieved in 62.9% of patients receiving 2.4 mg subcutaneously once weekly, compared with 34.3% in the placebo group (difference of 28.7 percentage points; 95% CI 21.1–36.2), while a reduction in fibrosis by at least one stage without worsening of MASH was observed in 36.8% versus 22.4% (difference of 14.4 percentage points; 95% CI 7.5–21.3). Both primary endpoints were met; a composite outcome was observed in 32.7% versus 16.1%, and the mean change in body weight was −10.5% versus −2.0% [417]. In August 2025, based on these data, the FDA granted the drug accelerated approval for the treatment of MASH with F2–F3 fibrosis; the final decision was deferred pending long-term clinical outcomes [418]. Two provisions of the updated American Association for the Study of Liver Diseases (AASLD) Practice Guidance are clinically significant: patient selection is recommended to be based on noninvasive tests rather than biopsy, and the combination of semaglutide with resmetirom has not been studied and therefore cannot be recommended [418].

In the Phase II SYNERGY-NASH study (*n* = 190, 52 weeks), the dual GIP and GLP-1 receptor agonist tirzepatide resulted in resolution of MASH without worsening fibrosis in 44%, 56%, and 62% of patients at doses of 5, 10, and 15 mg, compared with 10% in the placebo group; fibrosis improvement by at least one stage was observed in 55%, 51%, and 51% versus 30%, respectively [419]. An analysis at the individual-patient level clarified the nature of this response. Histological responders were characterized by greater body weight loss (−16.0% versus −7.0% for MASH resolution), a more significant reduction in HbA1c, and a more pronounced improvement in adipose tissue insulin sensitivity and adiponectin levels; normalization of liver fat content emerged as a statistically significant mediator of both steatohepatitis resolution and fibrosis improvement [420]. Thus, the effect of incretin therapy in MASH is largely mediated by correction of the “liver–adipose tissue” axis.

GLP-1 and glucagon receptor dual agonists demonstrate the greatest reduction in liver fat in absolute terms. In the Phase III SYNCHRONIZE-MASLD study (*n* = 216, 48 weeks), survodutide at a dose of 6.0 mg resulted in a reduction in liver fat content, as measured by MRI-PDFF, of at least 30% in 84.2% of patients compared with 24.3% in the placebo group, with changes in body weight of −12.2% versus −1.0% [421]. In a direct Phase IIa comparative study, efinopegdutide at a dose of 10 mg reduced liver fat content by 72.7% compared with 42.3% with semaglutide 1 mg over 24 weeks [422]. In the PROXYMO study, cotadutide reduced the hepatic fat fraction by 5.0 percentage points, ALT levels by 23.5 U/L, and AST levels by 16.8 U/L compared with placebo over 19 weeks in patients with biopsy-confirmed noncirrhotic MASH [423]. Dose-dependent gastrointestinal adverse events and the absence of histological endpoints in most of the studies conducted remain general limitations of this class.

Early studies with selective GLP-1 receptor agonists set the direction for the entire class. In the LEAN study, resolution of steatohepatitis was achieved within 48 weeks in 39% of patients receiving 1.8 mg of liraglutide, compared with 9% in the placebo group (RR 4.3; 95% CI 1.0–17.7), while fibrosis progression was observed in 9% versus 36% [424]. In the D-LIFT study, dulaglutide reduced liver fat content by 26.4% in relative terms over 24 weeks in patients with type 2 diabetes and NAFLD [425]. Retatrutide, a triple glucagon, GIP, and GLP-1 receptor agonist, has so far been evaluated only in preclinical models of obesity-associated MASH, where it caused a 31% reduction in body weight, a decrease in the homeostatic model assessment of insulin resistance (HOMA-IR) index, and a twofold reduction in liver triglyceride levels, without, however, improving the histological score in hamsters [426]. At the same time, clinical data for this drug have already been obtained: in a phase 2a study in patients with MASLD (*n* = 98), retatrutide, a triple GIP, GLP-1, and glucagon receptor agonist, reduced liver fat content measured by MRI-PDFF by 42.9%, 57.0%, 81.4%, and 82.4% at week 24 at doses of 1, 4, 8, and 12 mg, respectively, compared with a 0.3% increase in the placebo group (*p* < 0.001 for all doses). Liver fat content normalized to less than 5% in 79% and 86% of patients receiving 8 and 12 mg, respectively, compared with 0% in the placebo group [427]. Histological endpoints were not assessed, and by week 48, the number of participants with available magnetic resonance imaging (MRI) assessments had decreased to 8–9 per group, which limits the interpretation of long-term results [427].

Comparing treatment classes is difficult due to the lack of direct comparative studies. A network meta-analysis of 39 randomized trials (3311 participants), which used MRI-PDFF as a common denominator, ranked aldafermin, pegozafermin, and pioglitazone as the most effective treatments for absolute reduction in liver fat at 24 weeks, whereas efinopegdutide, the combination of semaglutide and firsocostat, and pegbelfermin ranked highest in terms of the proportion of patients achieving a reduction of at least 30% [428]. A systematic review assessing the certainty of evidence using the GRADE (Grading of Recommendations Assessment, Development and Evaluation) system, limited to studies in patients with biopsy-confirmed noncirrhotic MASH, confirms that resmetirom and incretin drugs act through different metabolic pathways and that no direct comparisons between them have been conducted [429]. The main characteristics of incretin-based drugs studied in MASLD and MASH are summarized in Table 3.

### 7.6. Targeting ApoC-III

Drugs that inhibit hepatic synthesis of ApoC-III have progressed from concept to approval more quickly than most drugs in the field of lipidology. In the placebo-controlled phase 3 BALANCE trial involving 66 patients with genetically confirmed FCS, olezarsen at a dose of 80 mg reduced triglyceride levels by 43.5 percentage points compared with placebo after 6 months, and ApoC-III levels by 73.7 percentage points. Over 53 weeks, 11 episodes of acute pancreatitis were reported in the placebo group, compared with one episode in each of the olezarsen groups [430]. On 19 December 2024, the drug received FDA approval for adult patients with FCS [431].

The siRNA plozasiran acts on the same target with a dosing frequency of once every three months. In the Phase III PALISADE trial involving 75 patients with persistent chylomicronemia, the median reduction in triglyceride levels after 10 months was 80% at the 25-mg dose and 78% at the 50-mg dose, compared with 17% in the placebo group. The incidence of acute pancreatitis also decreased [432]. Plozasiran was approved by the FDA in 2025 for the same indication [433,434]. A notable safety signal for the cardiometabolic population is hyperglycemia, which was observed in some patients with baseline prediabetes or diabetes mellitus [432].

The clinical development program extends beyond this rare phenotype. Olezarsen has been studied in patients with hypertriglyceridemia and high cardiovascular risk [435], as well as in those with moderate [436] and severe [437] hypertriglyceridemia. For plozasiran, it has been shown that ApoC-III inhibition alters not only the concentration but also the distribution of lipoprotein particles by size and number [438]. Clinical evaluation of ApoC-III inhibitors therefore focuses on two goals: preventing acute pancreatitis, particularly in patients with severe hypertriglyceridemia, and reducing residual cardiovascular risk through their effects on triglyceride-rich lipoproteins and remnant cholesterol [439]. In the context of MASLD, it is essential to distinguish between the site of action of the drug and the target of its clinical effect. Both groups of agents act in hepatocytes by inhibiting *APOC3* mRNA translation; however, the endpoint of their action lies in plasma—namely, the clearance of triglyceride-rich lipoproteins and remnant cholesterol. To date, no data are available on the effect of ApoC-III inhibition on the histological characteristics of MASH—steatosis, inflammation, and fibrosis. The clinical value of this approach for patients with MASLD lies in the fact that residual cardiovascular risk in steatotic liver disease is largely associated with remnant lipoproteins, and reducing this risk constitutes an independent therapeutic goal within the “liver–vasculature” axis [439].

### 7.7. Neurotransmitter Targets

Peripheral HTR2A blockade remains the best-justified yet least advanced of the strategies considered here. Experimental hepatic nerve blockade in mice slowed the development of diet-induced NAFLD while simultaneously lowering small intestinal serotonin content and the hepatic expression of HTR2A and lipogenic genes, and administration of an HTR2A antagonist reproduced this effect. Brain serotonin levels and HTR2C expression remained unchanged, which points to a peripheral localization of the target [179]. Combining a peripherally acting inhibitor of serotonin synthesis with a 5-HT2 receptor antagonist improved systemic insulin resistance in a model of glucocorticoid-induced metabolic dysfunction [440]. This line of investigation has not progressed beyond the preclinical stage. No peripherally restricted HTR2A antagonist has been evaluated in controlled trials in MASLD, and the 5-HT2A blockers available in clinical practice were developed for other indications and have not been optimized with respect to their central-to-peripheral exposure ratio. Yet it is precisely this ratio that determines whether the approach is practically feasible, since the therapeutic appeal of the target lies in the possibility of suppressing hepatic lipogenesis without affecting central serotonergic functions.

The dopaminergic direction presents the opposite situation. A drug exists and is in clinical use, but its hepatic effects have not been characterized directly. Bromocriptine-QR, administered within a narrow morning time window, normalizes the circadian peak of dopaminergic activity, reduces sympathetic tone, and attenuates the proinflammatory and pro-oxidative phenotype of peripheral blood mononuclear cells in patients with type 2 diabetes mellitus [231,441]. The dependence of this effect on the timing of administration has been confirmed experimentally: in spontaneously hypertensive rats maintained on a high-fat diet, bromocriptine corrected vascular pathology and manifestations of metabolic syndrome only when given during a specific phase of the circadian cycle [442]. No direct studies of the effect of bromocriptine on hepatic steatosis or fibrosis in humans have been conducted. The recently described D1R–Hippo/Yes-associated protein (YAP) axis warrants separate consideration. In rats with carbon tetrachloride–induced fibrosis, two weeks of levodopa administration reduced the proportion of collagen in liver tissue, increased D1R expression, and enhanced YAP phosphorylation, thereby suppressing its transcriptional activity [186]. Because YAP serves as a key mediator of hepatic stellate cell activation, the mechanism described links dopaminergic signaling directly to fibrogenesis [443].

Taken together, neurotransmitter targets represent the least mature segment of the therapeutic armamentarium considered here. Their value at this stage lies not in readiness for clinical application but in the way they broaden the concept of the regulatory architecture: hepatic lipogenesis and fibrogenesis prove amenable to modulation through neuronal and neuroendocrine inputs that operate independently of insulin, incretin, and thyroid hormone signaling.

### 7.8. Combination Strategies and Positioning in Clinical Practice

None of the approved agents acts simultaneously on all key nodes of pathogenesis. Resmetirom affects primarily intrahepatic lipid metabolism, incretin-based agents target systemic insulin resistance and adipose tissue dysfunction, and FGF21 analogs act on lipogenesis and fibrogenesis. This segregation of targets constitutes a direct rationale for combination therapy, and indirect comparisons support it. In a meta-analysis ranking interventions by the proportion of patients achieving a reduction in MRI-PDFF of at least 30%, the combination of semaglutide with the acetyl-CoA carboxylase inhibitor firsocostat was among the three most effective interventions [428]. An analysis that for the first time incorporated histological data on sodium-glucose cotransporter 2 (SGLT2) inhibitors and on newer combination regimens employing two or more agents with distinct mechanisms of action confirms the promise of a multitarget approach, demonstrating that drugs with different mechanisms of action can act complementarily on steatohepatitis and fibrosis [444]. Practical implementation, however, is outpacing the evidence base. The combination of resmetirom with semaglutide at a dose of 2.4 mg has not been examined in any trial [418], and questions of additivity of effect, summation of adverse events, and economic justification remain open. Cost-effectiveness analysis indicates that the choice between semaglutide and resmetirom in noncirrhotic MASH with moderate-to-advanced fibrosis (F2–F3) depends substantially on drug prices and treatment duration. Semaglutide shows more favorable cost-effectiveness metrics, particularly when its cardioprotective effects are taken into account, whereas the economic attractiveness of resmetirom is sensitive to price and duration of therapy [445].

Compensated cirrhosis remains a separate and as yet unresolved problem. In the SYMMETRY trial, in which efruxifermin was evaluated in 181 patients with cirrhosis due to MASH, the primary endpoint at week 36 was not met (a reduction in fibrosis of at least one stage without worsening of MASH: 19% with the 50-mg dose versus 13% with placebo); by week 96, however, the difference from placebo reached 16 percentage points (95% CI, 2 to 30) for the 50-mg dose [446]. This result illustrates a defining feature of the cirrhotic stage. Fibrosis regression requires substantially more time than standard protocols allow and becomes apparent only by week 96 of treatment, in contrast to the customary 36 to 48 weeks. This directly dictates the need to revise trial design and extend the duration of clinical trials in compensated cirrhosis [447,448].

Bioactive compounds of dietary origin warrant special consideration; interest in them stems from their effects on the same regulatory pathways discussed above. Accumulating experimental evidence suggests that bioactive substances derived from food may influence multiple epigenetic pathways involved in key hepatic and systemic processes, including lipid accumulation, redox balance, inflammatory signaling, mitochondrial activity, and the development of fibrosis. A diverse range of promising compounds—including newly investigated iridoid, certain ginsenosides, derivatives pentacyclic triterpene derivatives, ellagitannins, isoquinoline alkaloids, lignans, and other phenolics formulated [449] using advanced delivery approaches—has consequently attracted increasing therapeutic interest. While available clinical data are not yet adequate to justify their incorporation into standard treatment strategies, mechanistic and preclinical findings offer a scientific basis for further research into their potential role in MASLD and the “liver–other organs” axis management.

At the canonical level of regulation, berberine activates AMPK and reduces the expression of SREBP1 and FASN; in a combined protocol with metformin, these effects were partially reversed by an AMPK inhibitor [450]. In a high-fat diet model, berberine restored acetylation of histones H3 and H4 and reduced the repressive H3K9me3 mark at the *Cpt1a* locus—data that the authors explicitly state support, but do not prove, a chromatin mechanism [451]. These compounds are linked just as directly to non-canonical pathways. The protective effect of bilberry extract required intact FGF21/FGFR1c/β-Klotho signaling [452]. The effects of ginsenoside Rb1 were significantly attenuated in mice with adiponectin knocked out [453]. Chikusetsusaponin IVa acted as an inhibitor of lysine-specific demethylase 1 (LSD1), exerting its effect via the intestinal FXR–small heterodimer partner (SHP) axis [454]. Clinical trials, however, clearly demonstrate a disconnect between surrogate and imaging endpoints. A meta-analysis of ten randomized controlled trials involving 811 patients showed a reduction in ALT activity and the HOMA-IR index with berberine supplementation [455], whereas in 337 patients with obesity and MASLD, six months of taking 1 g of berberine daily did not alter either liver fat content (0.9%; 97.5% CI from −0.4 to 2.1) or visceral fat area, despite an improvement in the lipid profile [456]. The most encouraging results were obtained with curcumin. Over 24 weeks, it reduced the CAP by 17.5 dB/m (95% CI from −27.1 to −7.8) while altering the composition of the microbiota, increasing deoxycholic acid (DCA) levels, TGR5 expression, and GLP-1 levels—that is, it acted along the “gut-liver” axis [457]. At the same time, a Cochrane review of 17 randomized controlled trials involving 2069 patients rates the certainty of evidence for silymarin as very low (a reduction in ALT activity of 7.21 U/L; 95% CI from −10.62 to −3.80), with a complete lack of data on mortality and quality of life [458], while a meta-analysis of 131 randomized trials (*n* = 9191) showed that, despite a noticeable reduction in transaminases, a significant decrease in liver stiffness was achieved only with curcumin (MD −0.76; *p* = 0.03) and pomegranate (MD −0.86; *p* = 0.04), although the dosage forms were highly heterogeneous [459]. Dietary bioactive compounds can rightly be considered tools for studying canonical and non-canonical pathways and as a potential adjunct to dietary interventions, but not as a component of standard MASLD therapy.

## 8. Limitations and Unresolved Questions

The present review is a narrative review and did not involve a systematic search conducted under a prospectively registered protocol; sources were selected according to criteria of topical relevance and level of evidence, with priority given to randomized trials, meta-analyses, and recent mechanistic studies. The picture presented accordingly reflects the state of the field but is not free of publication bias.

The most substantial limitation of the evidence base is translational. A considerable portion of the mechanistic conclusions regarding non-canonical regulators derives from rodent studies, whereas interspecies differences in this domain are unusually pronounced. Leptin deficiency in mice prevents fibrosis in the setting of chronic liver injury, whereas in pigs it reproduces the full spectrum of MASLD up to advanced fibrosis. Similarly, the concept of FGF21 resistance rests primarily on data showing reduced expression of β-Klotho and FGFR1c in rodent tissues, while direct evidence of tissue-level resistance in humans remains scant. At the same time, recent genetic approaches, Mendelian randomization in particular, provide compelling evidence of substantial pleiotropy in FGF21 signaling that extends well beyond lipid metabolism and encompasses effects on alcohol consumption, the risk of alcohol-related liver disease, and other phenotypes [460].

A second limitation relates to biomarkers. Neither FGF21 nor adiponectin has been validated for risk stratification in CKM syndrome. Large prospective studies evaluating these measures as predictors of transition between CKM stages have not been performed, and the direction of their changes at advanced disease stages is inconsistent, complicating interpretation. Noninvasive fibrosis tests and patient referral pathway models remain more mature in practice [461,462], whereas incorporation of non-canonical mediators into diagnostic algorithms remains premature.

A third group of limitations relates to clinical trial methodology. Histological endpoints in MASH are subject to sampling variability [463,464] and interobserver disagreement [464,465], and the magnitude of response in placebo groups varies widely, depending on the characteristics of the enrolled population and on features of the protocol [466]. Tellingly, the rate of steatohepatitis resolution without worsening of fibrosis in placebo groups can differ dramatically even between methodologically similar trials. In the phase 3 ESSENCE trial, it was 34.3% in patients with F2–F3 fibrosis at 72 weeks (difference vs. semaglutide 2.4 mg, 28.7 percentage points; 95% CI, 21.1 to 36.2) [417], whereas in the phase 2b SYNERGY-NASH trial of tirzepatide it was only 10% at week 52 [419]. Such variability in the placebo response highlights the impact of the characteristics of the enrolled population, the duration of the intervention, and features of the protocol, precluding direct comparison of absolute values across trials and requiring caution in constructing indirect comparisons.

Finally, MASLD is not a homogeneous disease entity. Genetic variants in *PNPLA3*, *TM6SF2*, *MBOAT7*, and *HSD17B13* determine substantially divergent disease trajectories and, in all likelihood, differing responses to interventions directed at the non-canonical axes [467], while sex- and age-related differences in the regulation of hepatic lipid metabolism are mediated at the level of transcriptional programs [468] and are discussed in detail in Section 4.5. None of the therapeutic approaches considered to date has been studied with such stratification, and presumably, it is precisely here that the greatest opportunity to improve treatment efficacy lies.

## 9. Conclusions

The data presented here show that hepatic lipid metabolism is governed not only by insulin and the canonical transcriptional programs under its control, but also by an extensive network of non-canonical regulators—the fibroblast growth factors FGF21 and FGF19, adipokines, apolipoprotein C-III, and monoamine neurotransmitters. These molecules operate within the liver–adipose tissue, gut–liver, and brain–liver interorgan axes, coordinating lipolysis, lipogenesis, lipoprotein secretion, inflammation, and fibrogenesis. Disruption of the dynamic balance between canonical and non-canonical signals constitutes a key mechanism of the transition from simple steatosis to steatohepatitis and fibrosis, and it accounts for the clinical heterogeneity of MASLD.

The degree of clinical readiness differs across these approaches. Two agents have obtained regulatory approval for noncirrhotic MASH with stage F2–F3 fibrosis: the thyroid hormone receptor-β agonist resmetirom (March 2024) and the GLP-1 receptor agonist semaglutide (August 2025). Analogs of FGF21 and FGF19, along with dual and triple incretin agonists, are in phase 2 and phase 3 clinical development. Agents directed against ApoC-III have been approved, but for a different indication, and their relevance to MASLD is mediated by the reduction in cardiometabolic risk. Adiponectin receptor agonists, resensitizers of leptin signaling, and neurotransmitter targets remain a preclinical prospect.

Further progress will most likely be determined by three lines of development. The first is the integration of non-canonical biomarkers into risk stratification algorithms within the cardiovascular–kidney–hepatic–metabolic framework. This requires prospective validation, which has not yet been undertaken. The second is combination regimens acting simultaneously on several interorgan axes. Their rationale follows from the nonoverlapping mechanisms of action of the approved agents, yet it has been supported only by indirect comparisons. The third is organ-selective targeting of peripheral targets, primarily of hepatic HTR2A and the adiponectin receptors, which could potentially achieve a metabolic effect with minimal systemic and central adverse events. The expansion of the insulin-centric paradigm into a model of an interorgan regulatory network represents not a theoretical superstructure but a working basis for the selection of therapeutic targets—a proposition confirmed by the clinical success of the first agents acting outside the insulin cascade. A comparison of the data presented reveals a principle that is important for planning future research: the outcome of an intervention is determined not only by the direction of change in a lipid metabolite but also by the tissue and the specific cell in which this change occurs. Therefore, a reduction in liver fat content should not be equated with an antifibrogenic effect, and the evaluation of new interventions requires separate consideration of lipid fluxes, hepatocyte damage, and extracellular matrix turnover—taking into account sex, reproductive status, and the immune component of interorgan regulation.

## Figures and Tables

**Figure 1:**
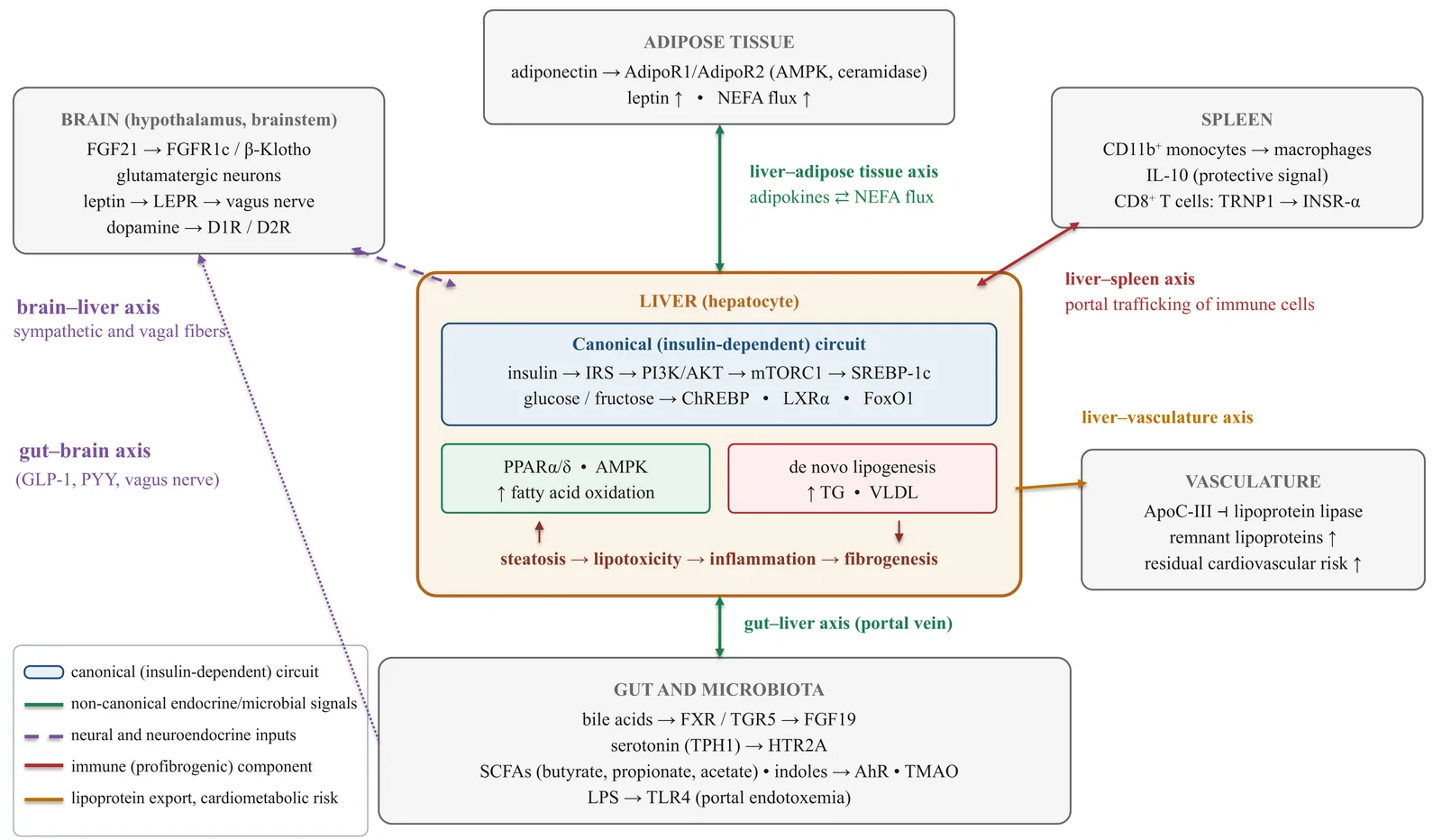
Canonical and non-canonical pathways regulating hepatic lipid metabolism and interorgan axes. The canonical pathway (blue panel) integrates the insulin-dependent IRS—PI3K/AKT—mTORC1—SREBP-1c pathway and the nutrient-dependent ChREBP branch; the oppositely directed β-oxidation program is regulated by PPARα/δ and AMP-activated protein kinase (AMPK). Non-canonical signals (green) reach the liver via the “liver–adipose tissue” axis (adiponectin via AdipoR1/2, leptin, and the flow of non-esterified fatty acids (NEFAs)) and the “gut–liver” axis (bile acids via farnesoid X receptor (FXR) and Takeda G protein-coupled receptor 5 (TGR5) with FGF19 induction, serotonin via HTR2A, short-chain fatty acids, indole metabolites, trimethylamine-N-oxide, and lipopolysaccharide via TLR4). Neuronal and neuroendocrine inputs (purple) form the “brain–liver” axis: the central action of FGF21 on glutamatergic neurons, the vagal component of leptin’s effect, and dopaminergic regulation via dopamine receptors D1R and D2R, as well as the ascending “gut–brain” pathway. The immune component (red) is represented by the “liver–spleen” axis: portal transport of monocytes, the protective action of interleukin-10, and the profibrogenic signaling of CD8^+^ T cells via the soluble α-subunit of the insulin receptor. The “liver–vasculature” axis (orange) reflects the export of lipoproteins, during which ApoC-III inhibits lipoprotein lipase and increases the pool of remnant particles. The result of the integration of these inputs determines the transition from steatosis to lipotoxic damage, inflammation, and fibrogenesis. Colored lines denote the interorgan signal routes listed in the legend; gray panels identify organs and tissues, and the shading of the two panels within the hepatocyte distinguishes the opposing programs of fatty acid oxidation (green) and de novo lipogenesis (red).

**Table 1:** Non-canonical regulators of hepatic lipid metabolism and their interorgan axes.

Regulator	Source	Receptor/Target	Interorgan Axis	Effect on Hepatic Lipids
FGF21	Liver (hepatocytes)	FGFR1c/β-Klotho	Liver–adipose tissue; liver–brain	↓ steatosis, ↑ β-oxidation, ↓ fibrogenesis
FGF19/FGF15	Ileum	FGFR4/β-Klotho	Gut–liver	↓ bile acid synthesis, ↓ lipogenesis and gluconeogenesis
Adiponectin	Adipose tissue	AdipoR1/R2 → AMPK, ceramidase	Liver–adipose tissue; liver–kidney	↓ lipogenesis, ↑ oxidation, ↓ inflammation and fibrosis
Leptin	Adipose tissue	LepR (CNS, stellate cells)	Brain–liver; liver–adipose tissue	Centrally, an antisteatotic effect; in hyperleptinemia and leptin resistance, potential enhancement of inflammation and fibrogenesis
ApoC-III	Liver, intestine	LPL inhibition	Liver–vasculature	↑ VLDL secretion, ↑ plasma triglycerides
Serotonin	Intestinal enterochromaffin cells	HTR2A	Gut–liver	↑ lipogenesis, ↑ steatosis, ↑ ER stress
Dopamine	CNS, peripheral tissues	D1R/D2R	Brain–liver	Modulation of insulin signaling, ↓ glucose levels
Splenic immune mediators (IL-10, monocytes, CD8^+^ T cells)	Spleen	IL-10R; soluble α-subunit of the insulin receptor → ERK—p90RSK in stellate cells	Liver—spleen	A dual effect: IL-10 limits triglyceride accumulation and inflammation; monocyte recruitment and CD8^+^ T-cell signaling enhance fibrogenesis

Abbreviations: AdipoR1/R2, adiponectin receptors 1 and 2; AMPK, AMP-activated protein kinase; ApoC-III, apolipoprotein C-III; CNS, central nervous system; D1R/D2R, dopamine receptors D1 and D2; ER, endoplasmic reticulum; ERK, extracellular signal-regulated kinase; FGF15/FGF19/FGF21, fibroblast growth factor 15/19/21; FGFR1c/FGFR4, fibroblast growth factor receptor 1c/4; β-Klotho, beta-klotho co-receptor; HTR2A, 5-hydroxytryptamine (serotonin) receptor 2A; IL-10, interleukin-10; IL-10R, interleukin-10 receptor; LepR, leptin receptor; LPL, lipoprotein lipase; p90RSK, p90 ribosomal S6 kinase; VLDL, very-low-density lipoprotein. Arrows: ↑, increase; ↓, decrease; →, signaling/pathway activation.

**Table 2:** Stage-specific changes in canonical and non-canonical regulators in MASLD.

Regulator or Signal	Simple Steatosis	Steatohepatitis	Fibrosis	Principal Mechanism and Limitations	References
De novo lipogenesis (contribution to the hepatic palmitate pool)	↑ (19% in obesity without steatosis → 38% in MASLD)	↑↑ (40.7%)	Remains elevated (36.8% in cirrhosis); not associated with liver stiffness	Substrate-driven activation of ChREBP; “selective” hepatic insulin resistance has not been confirmed in humans	[41,260,261]
Nonesterified fatty acid influx	Basal lipolysis is not elevated; insulin-mediated suppression is impaired (more than sixfold higher insulin requirement)	↑ With worsening adipose tissue insulin resistance	Adipo-IR is independently associated with fibrosis (OR, 1.51; 95% CI, 1.05–2.16)	Loss of the antilipolytic effect of insulin; cross-sectional evidence does not establish causality	[263,266,267]
Triglyceride export in VLDL	↑ Approximately twofold	Plateaus when intrahepatic lipid content exceeds 10%	Inversely correlated with lipid content (r = −0.92)	Saturation of export capacity: the increase in export does not match influx	[268,269]
Adiponectin	↓	↓↓ (WMD relative to simple steatosis, 1.81; 95% CI, 1.09–2.53)	No difference detected between patients with and without fibrosis (SMD, −0.15; 95% CI, −0.35 to 0.05)	True ligand deficiency; Mendelian randomization studies do not support a causal role in insulin resistance	[286,298,314]
FGF21	↑	↑↑ (386.6 ± 328.9 vs. 297.9 ± 231.5 pg/mL; *p* = 0.009)	↑ With increasing severity of liver injury	Tissue resistance despite ligand excess; these changes are a consequence of the disease rather than its cause	[88,284,296]
Leptin	↑ (SMD, 0.36 vs. controls)	↑↑ (SMD, 0.21 vs. simple steatosis)	Direct profibrogenic effects on hepatic stellate cells	Central resistance with peripheral profibrogenic effects; genetically elevated levels are associated with lower risk	[285,297,315]
FGF19	↓	↓ With suppression of FXR and FGFR4 signaling	ND	Ligand deficiency in the setting of an altered bile acid profile	[88,287]
Gut-derived serotonin (HTR2A)	↑ Lipogenesis	↑ Inflammation and endoplasmic reticulum stress	ND in humans	Gut–liver axis; evidence is predominantly experimental	[173,174,175]
Microbial metabolites (acetate, indoles, trimethylamine N-oxide)	Acetate serves as a substrate for lipogenesis	↑ Proinflammatory contribution; elevated trimethylamine N-oxide (SMD, 0.66)	Lower indole-3-propionic acid levels in fibrosis (*p* = 0.039)	Substrate-mediated and receptor-mediated pathways (Section 4.2); substantial heterogeneity in estimates	[221,224]
Ceramides and sphingosine-1-phosphate	↑ In parallel with steatosis	↑ (hepatic ceramide levels are 50% higher than in simple steatosis)	Sphingosine-1-phosphate promotes fibrosis through S1PR2 without affecting steatosis	Cell-specific effects: the direction of change depends on cell type (Section 3.5)	[194,197]
Immune niches (TREM2^+^ macrophages, lipid-associated macrophages)	Emerge in steatotic regions	Accumulation of TREM2^+^S100A9^+^ cells and monocytic myeloid-derived suppressor cells	Scar-associated niches with ACKR1^+^ and PLVAP^+^ endothelial cells	Context-dependent and stage-specific roles: TREM2 deficiency exacerbates steatohepatitis	[306,307,308]
Hepatic stellate cells and extracellular matrix turnover	Quiescence	Activation of stellate cell subclusters that constitute the main source of matrix	Core activation-associated genes (SERPINE1, GAS7, SPON1, LTBP2)	Fibrosis regression requires enhanced matrix degradation, not merely suppression of matrix synthesis	[312,316]
Splenic immune input	↑ After splenectomy, but only with a high-fat diet, not a normal diet	Loss of IL-10-dependent protection exacerbates steatosis and inflammation	CD8^+^ T cells with high TRNP1 expression activate hepatic stellate cells through INSR-α	Liver–spleen axis (Section 4.4); evidence is predominantly experimental	[239,240,242]

Abbreviations: ACKR1, atypical chemokine receptor 1; Adipo-IR, adipose tissue insulin resistance index; CD8, cluster of differentiation 8; ChREBP, carbohydrate responsive element-binding protein; CI, confidence interval; FGF19 and FGF21, fibroblast growth factors 19 and 21, respectively; FGFR4, fibroblast growth factor receptor 4; FXR, farnesoid X receptor; GAS7, growth arrest specific 7; HTR2A, 5-hydroxytryptamine (serotonin) receptor 2A; IL-10, interleukin-10; INSR-α, soluble insulin receptor α subunit; LTBP2, latent transforming growth factor beta binding protein 2; MASLD, metabolic dysfunction-associated steatotic liver disease; ND, no data available; OR, odds ratio; PLVAP, plasmalemma vesicle-associated protein; S100A9, S100 calcium-binding protein A9; S1PR2, sphingosine-1-phosphate receptor 2; SERPINE1, serpin family E member 1; SMD, standardized mean difference; SPON1, spondin 1; TREM2, triggering receptor expressed on myeloid cells 2; TRNP1, TMF1-regulated nuclear protein 1; VLDL, very-low-density lipoprotein; WMD, weighted mean difference. Note: ↑ and ↓ indicate increases and decreases, respectively; double arrows indicate more pronounced changes and do not specify fold changes. → indicates a transition between the stated groups; ^+^ indicates marker-positive cells.

**Table 3:** Incretin drugs in clinical and preclinical studies in MASLD/MASH.

Drug	Mechanism of Action	Phase, Trial	Key Results	Adverse Events and Safety Considerations	Duration, Weeks	References
Semaglutide 2.4 mg	GLP-1 receptor agonist	Phase 3, ESSENCE (NCT04822181)	Resolution of MASH without worsening of fibrosis 62.9% vs. 34.3%; reduction in fibrosis 36.8% vs. 22.4%; body weight −10.5% vs. −2.0%	Predominantly gastrointestinal events: nausea, 36.3% vs. 13.2%; diarrhea, 26.9% vs. 12.2%; vomiting, 18.6% vs. 5.6%. Discontinuation due to adverse events: 2.6% vs. 3.3%; acute pancreatitis: 0.4% vs. 0.5%	72	[417,429]
Tirzepatide 5/10/15 mg	Dual GIP and GLP-1 receptor agonist	Phase 2, SYNERGY-NASH (NCT04166773)	Resolution of MASH 44/56/62% vs. 10%; reduction in fibrosis 55/51/51% vs. 30%	Nausea: 36.2/34.0/43.8% vs. 12.5%; diarrhea: 31.9/36.2/27.1% vs. 22.9%; decreased appetite: 19.1/21.3/22.9% vs. 2.1%; serious adverse events: 10.6/8.5/0% vs. 6.3%. Isolated cases of pancreatitis and cholecystitis	52	[419]
Survodutide 6.0 mg	Dual glucagon and GLP-1 receptor agonist	Phase 3, SYNCHRONIZE-MASLD	MRI-PDFF reduction ≥ 30% in 84.2% vs. 24.3%; body weight −12.2% vs. −1.0%	Nausea: 56.2% vs. 14.3%; vomiting: 42.5% vs. 7.1%; diarrhea: 29.5% vs. 10.0%. Treatment discontinuation due to adverse events: 23.3% vs. 10.0%, predominantly during dose escalation	48	[421]
Efinopegdutide 10 mg	Dual glucagon and GLP-1 receptor agonist	Phase 2a (NCT04944992), active comparator: semaglutide 1 mg	Reduction in hepatic fat content of 72.7% vs. 42.3%	Adverse events: 79.2% vs. 60.3% (semaglutide, 1 mg); constipation: 16.7% vs. 5.5%; abdominal pain: 12.5% vs. 2.7%. Serious adverse events: 1.4% in both groups	24	[422]
Cotadutide 300/600 µg	Dual glucagon and GLP-1 receptor agonist	Phase 2a, PROXYMO (NCT04019561)	Hepatic fat fraction −5.0 percentage points; ALT −23.5 U/L; AST −16.8 U/L (600 µg dose vs. placebo)	Adverse events: 91.7% (600 μg) and 76.9% (300 μg) vs. 37.5%, predominantly mild or moderate gastrointestinal events. Treatment discontinuation: 16.7% and 7.7%, respectively, vs. 4.2%	19	[423]
Liraglutide 1.8 mg	GLP-1 receptor agonist	Phase 2, LEAN (NCT01237119)	Resolution of steatohepatitis 39% versus 9%; fibrosis progression 9% vs. 36%	Gastrointestinal disorders: 81% vs. 65%; diarrhea: 38% vs. 19%; constipation: 27% vs. 0%; decreased appetite: 31% vs. 8%. Events were predominantly grade 1–2 and transient	48	[424]
Dulaglutide 1.5 mg	GLP-1 receptor agonist	D-LIFT RCT	Relative reduction in hepatic fat content of 26.4%; reduction in GGT and transaminase activity	No serious drug-related adverse events were reported; detailed frequencies of gastrointestinal symptoms were not published	24	[425]
Retatrutide	Triple glucagon, GIP, and GLP-1 receptor agonist	Preclinical MASH models (mice and hamsters); phase 2a MASLD substudy (NCT04881760)	Preclinical: body weight, −31%; reduced HOMA-IR; hepatic triglycerides, −50%. Clinical (phase 2a): liver fat reduced by 81.4% (8 mg) and 82.4% (12 mg) at week 24; normalization (<5%) in 79% and 86% of patients, respectively	Preclinical models: reduced food intake with loss of both fat and lean mass. The phase 2a MASLD substudy predominantly reported transient gastrointestinal events; serious adverse events occurred in 2.5% (including acute cholecystitis); no hepatotoxicity signals were identified	—	[426,427]

Abbreviations: ALT, alanine aminotransferase; AST, aspartate aminotransferase; D-LIFT, Effect of Dulaglutide on Liver Fat; GGT, gamma-glutamyl transferase; GIP, glucose-dependent insulinotropic polypeptide; GLP-1, glucagon-like peptide-1; HOMA-IR, homeostatic model assessment of insulin resistance; LEAN, Liraglutide Efficacy and Action in Nonalcoholic Steatohepatitis; MASH, metabolic dysfunction-associated steatohepatitis; MASLD, metabolic dysfunction-associated steatotic liver disease; MRI-PDFF, magnetic resonance imaging-derived proton density fat fraction; NASH, nonalcoholic steatohepatitis; NCT, National Clinical Trial identifier (ClinicalTrials.gov registration number); RCT, randomized controlled trial; U/L, units per liter.

## Data Availability

No new data were created or analyzed in this study. Data sharing is not applicable to this article.
